# Meta-analysis of the predictive value of critical care echocardiography for weaning outcomes in patients with VA-ECMO-assisted cardiogenic shock

**DOI:** 10.3389/fmed.2026.1835564

**Published:** 2026-06-25

**Authors:** Miao Niu, Yongshun Feng

**Affiliations:** Beijing Jingmei Group General Hospital, Beijing, China

**Keywords:** cardiogenic shock, critical care echocardiography, meta-analysis, venoarterial extracorporeal membrane oxygenation, weaning prediction

## Abstract

**Objective:**

This study aimed to evaluate the predictive value of critical care echocardiographic parameters for weaning outcomes in patients receiving venoarterial extracorporeal membrane oxygenation (VA-ECMO) for cardiogenic shock.

**Methods:**

A comprehensive literature search was conducted across PubMed, Embase, Cochrane Library, Web of Science, Scopus, and CINAHL from inception to December 2025. Studies reporting echocardiographic predictors of VA-ECMO weaning outcomes were included. A two-component analytical approach was applied, which pooled standardized mean differences for association analyses and used bivariate random-effects modeling (Reitsma method) for diagnostic accuracy.

**Results:**

A total of 37 studies encompassing 3,458 patients were analyzed. Aortic valve opening status exhibited the highest discriminative ability (AUC = 0.88, 95% CI: 0.82–0.93), followed by left ventricular outflow tract velocity-time integral (AUC = 0.85, 95% CI: 0.81–0.88), tissue Doppler-derived mitral annular systolic velocity (AUC = 0.81, 95% CI: 0.76–0.86), and left ventricular ejection fraction (AUC = 0.79, 95% CI: 0.75–0.83). Provisional reference thresholds, derived primarily from studies using a 48-h decannulation definition, included LVEF 20–25%, LVOT-VTI ≥ 10 cm, and TAPSE ≥17 mm; these should be interpreted as exploratory rather than confirmatory.

**Conclusion:**

This meta-analysis describes a hierarchy of echocardiographic parameters for predicting VA-ECMO weaning success and provides provisional reference thresholds that may inform—but do not replace — individualized clinical judgment in cardiogenic shock management.

## 1 Introduction

Cardiogenic shock is the most severe form of acute cardiac dysfunction, which is distinguished by a severe reduction in cardiac output that underlies a spectrum of systemic hypoperfusion and multi-organ failure (1). Despite a great deal of progress that has been achieved in drug therapy and mechanical cardiac support for the past few decades, the rate of mortality remains unacceptably high, between 40 to 50%, which attests to the clinical challenge that this life-threatening illness poses (2). Venoarterial extracorporeal membrane oxygenation (VA-ECMO) is an important emergency and temporary form of mechanical support for patients with refractory cardiogenic shock. VA-ECMO provides total cardiopulmonary bypass and helps to facilitate cardiac recovery or bridging to final therapies such as ventricular assist devices and heart transplants (3). According to the Extracorporeal Life Support Organization database, there has been a significant increase in VA-ECMO utilization in recent years, with a tenfold increase in adult cardiac ECMO utilization on an annual basis. This reflects its important role in managing cardiogenic shock (4).

Professional society guidance—including the JACC Scientific Expert Panel on VA-ECMO (5), the AHA Scientific Statement on escalation and de-escalation of temporary mechanical circulatory support (6), the ASE Updated Recommendations for imaging during temporary mechanical support (7), and the pragmatic weaning framework proposed by Randhawa et al. (8)—consistently emphasizes individualized, multiparametric approaches to liberation from mechanical support, yet a synthesis integrating both diagnostic accuracy and association data remains absent from the literature. The major therapeutic goal in VA-ECMO support is to have a successful weaning through native recovery. The most challenging aspect in ECMO management is to determine when to withdraw support successfully. Early stoppage of mechanical circulatory support is prone to acute hemodynamic failure and urgent reinitiation of ECMO, while prolonged support is prone to various complications related to the use of ECMO, which include bleeding complications, thromboembolism, infections, ischemia of limbs, and hemolysis (9). Registry studies and systematic reviews have shown that successful weaning rates have been reported anywhere between 31 and 76%, depending on etiology. A large number of patients who successfully gained weaning also did not survive to be discharged home (10). These figures highlight the key need for reliable predictive models to accurately detect which patients show adequate recovery of the myocardium to be discharged from assistance as soon as possible without delaying a discharge that is premature.

Critical care echocardiography has now been recognized as the core imaging modality for the assessment of cardiac recovery in the context of VA-ECMO, as it satisfies the particular needs of intensive care with bed-side, real-time imaging (11). Numerous intensive care variables in echocardiography have been investigated for potential prediction of successful weaning, each estimating a different physiological element of cardiac recovery. The Left ventricular ejection fraction provides a generalized assessment of cardiac contractile capability and thus far is the best evaluated variable, suggesting a restored contractile ability to maintain self-sufficiency in circulation (12). Left ventricular outflow tract velocity-time integral (LVOT-VTI) directly reflects stroke volume and forward cardiac output, with values exceeding 10 cm generally considered indicative of adequate systemic perfusion. Tissue Doppler-derived mitral annular systolic velocity offers relatively load-independent evaluation of longitudinal contractile function. The landmark study by Aissaoui et al. (13) established the foundational echocardiographic criteria for VA-ECMO weaning using a standardized minimal-flow trial protocol, during which patients underwent haemodynamic challenge at reduced ECMO support with simultaneous echocardiographic assessment. Patients who maintained haemodynamic stability during this flow-reduction trial and demonstrated LVEF exceeding 20–25% and aortic VTI exceeding 10 cm were found to tolerate successful weaning, establishing these values as the most widely cited provisional thresholds in subsequent literature. Subsequent studies have gradually expanded this list of potential predictive biomarkers to include aortic valve opening status as a surrogate predictor for left ventricular pressure development, right ventricular to pulmonary artery coupling indices to incorporate the crucial function of right heart function (14), or right ventricular ejection fraction measured by three-dimensional critical care echocardiography as a volumetric measure of right ventricular function.

However, despite the growing number of studies being conducted on critical care echocardiographic predictors for VA-ECMO weaning outcomes, there are still challenges in the current body of evidence that make translation to clinical practice difficult. The greatest body of studies is retrospective in nature, conducted in single institutions, with small sample populations, which might introduce bias. There is also variability in the definition of successful weaning, which might range from 24 h to hospital discharge in different studies. Regarding the time and hemodynamic conditions of the critical evaluation of echocardiographic examination, there is great variability among individuals who get measurements at baseline as well as during weaning trials performed at different levels of decreased flow or before actual decannulation. In reality, a clearly defined protocol of evaluation has not yet been established. Each study uses different cut-offs for different parameters. Whereas there are many previous studies that have explored the predictive utility of critical care echocardiography in relation to various parameters using association measures (odds ratio, for instance) or diagnostic accuracy measures (sensitivity, specificity), to the best of our knowledge no previous syntheses have incorporated both measures in their analysis. Lack of meta-analysis of diagnostic accuracy measures such as summary sensitivity, specificity, and area under summary ROC curves is clearly a limitation of previous research. Several previous studies have explored aspects of this issue. Burgos et al. (11) pooled multiparametric predictors but did not include a formal diagnostic-accuracy synthesis. Charbonneau et al. (9) summarized parameters qualitatively. Hsu et al. (15) published the most recent meta-analysis involving prospective study registration, but concentrated their efforts mainly on correlation coefficients. The current review represents an expansion of the existing literature by including studies that presented sophisticated parameters (3D RVEF, LA strain, total isovolumic time), using bivariate modeling of diagnostic test accuracy to calculate sensitivities, specificities, and AUC for each parameter, and summarizing cut-off values.

The purpose of this systematic review and meta-analysis is to fill this gap by systematically assessing the ability of echocardiographic critical care parameters as predictors of weaning outcome among patients undergoing VA-ECMO with cardiogenic shock. As a continuation of previous systematic reviews, which have mostly focused on association statistics, the present research uses an innovative analytical method that combines two statistical models: standardized mean difference pooling and bivariate random effects diagnostic accuracy meta-analyses (16). Although the use of such models is well-documented in other diagnostic fields, to the best of our knowledge this approach has not been used previously for the assessment of VA-ECMO weaning in this particular context. Therefore, the significance of this research lies not solely in its methodology but in its unique application to this clinical context. Specifically, the present study offers a parameter-level analysis by summarizing the area under the curve of each variable. Additionally, it offers an informative compilation of all reported cut-off values in the literature. Such a compilation can be beneficial for clinical practice and might aid in developing critical care echocardiographic-guided weaning protocols.

## 2 Methods

### 2.1 Protocol and registration

This study was conducted according to the PRISMA 2020 guidelines for systematic reviews and meta-analyses and the PRISMA-DTA statement extension for diagnostic test accuracy studies. This study was not registered in the PROSPERO database; this was recognized as a limitation of the study design. To account for this limitation and prevent potential bias related to selective reporting of data, the entire *a priori* protocol was available as Supplementary File 1 and could be accessed together with this manuscript. This protocol included research questions, inclusion criteria, search strategy, extracted variables, predetermined analyses, and subgroup and sensitivity analyses. All analyses conducted within this study were pre-specified in the protocol; any additional analyses introduced on the basis of peer reviewer comments were clearly identified as post-hoc analyses. A complete search strategy can be found in Supplementary Table 1. All subgroup and sensitivity analyses had been pre-specified in the protocol; results of all analyzed parameters were provided irrespective of their statistical significance. Since this study did not involve direct work with humans, ethical approval was not required.

### 2.2 Eligibility criteria

Selection criteria for this systematic review were based on modifications of the PIRD framework applied to diagnostic accuracy research. Inclusion criteria involved adult patients aged 18 years or older treated with venoarterial ECMO for cardiogenic shock of various etiologies, such as myocardial infarction, acute decompensated heart failure, post-cardiotomy shock, and fulminant myocarditis. Pediatric populations were excluded because of different hemodynamic features. Research that focused on venovenous ECMO for respiratory failure or extracorporeal cardiopulmonary resuscitation was also excluded due to dissimilarities in clinical scenarios and objectives. As part of the index test, the following VA-ECMO-associated variables were relevant to the diagnostic accuracy test: critical care echocardiographic measurements such as left ventricular ejection fraction, left ventricular outflow tract velocity time integral, aortic valve opening, tissue Doppler mitral annular systolic velocity, tricuspid annular plane systolic excursion, and right ventricular fractional area change.

The principal reference standard for weaning success was decannulation from VA-ECMO without any reintubation or initiation of a durable mechanical circulatory assist device for at least 48 h after decannulation. Considering that many of the eligible trials had used a different reference standard, it was decided that the primary analysis would be stratified on the basis of a specific reference standard, as follows: (1) 48-h survival after decannulation without any reintubation (primary reference standard); (2) 24-h survival after decannulation; and (3) hospital discharge. Pooled estimates were provided separately for all three strata.

Prospective or retrospective cohort studies, case–control studies, or diagnostic accuracy studies that provided an association measure between critical care echocardiography parameters and weaning were considered eligible for inclusion in the meta-analysis. Excluded studies were case series of fewer than ten patients, review articles, editorials, and conference abstracts when no full-text article was available.

### 2.3 Information sources and search strategy

The literature search was conducted across multiple electronic databases to ensure comprehensive identification of relevant studies, including PubMed/MEDLINE, Embase, Cochrane Central Register of Controlled Trials, Web of Science Core Collection, Scopus, and CINAHL. Clinical trial registries including ClinicalTrials.gov and the International Clinical Trials Registry Platform were also searched to identify completed but unpublished studies and ongoing research in this field. This search strategy included the use of terms in a controlled vocabulary combined with free-text search terms in a set of conceptual topic areas including extracorporeal membrane oxygenation and related terms for mechanical support, cardiogenic shock and related terms for hemodynamic compromise, weaning and liberation from support, and finally, critical care echocardiography and related imaging techniques. The complete search strategy can be found in Supplementary Table 1. Language restrictions were not used. The first search was performed December 1–15, 2025. To keep the information current prior to resubmission, an update search was performed using the same search strategy, without any further studies being identified that would affect the meta-analysis results. The bibliographies from the selected trials and the systematic reviews were reviewed manually for any other possible literature sources.

### 2.4 Study selection

The study selection was done through a two-step screening process carried out independently by two reviewers (M.N. and Y.S.F.). To promote agreement between the reviewers in assessing the records for eligibility prior to the formal screening process, a calibration exercise using a random sample of 50 records with a target inter-rater agreement, as measured by Cohen’s kappa, of 0.80 or higher was implemented. In the first stage of study selection, the title and abstract were screened against the specified study selection criteria. The articles that qualified from the initial screening proceeded to the full-text screening for validation and harvesting of the relevant information. Any discrepancies that arose during the screening process for articles that did not make the final cut were resolved by consultation and consensus. The justification for exclusion during the full-text screening was as per the PRISMA systematic review guidelines.

### 2.5 Data extraction

A standardized data extraction sheet, which was first tested on a selected number of eligible studies, was used to extract all relevant data for meta-analysis. The details of the study were obtained from each study, such as authorship, year of publication, location of the study, study design, duration of enrollment, and total number of subjects. The population-related details included patient characteristics, etiology of cardiogenic shock, disease severity, ECMO mode (whether peripheral or central cannulation was used), duration of support, and—importantly—associated LV unloading technique, which included the use of intra-aortic balloon pump (IABP), Impella (a percutaneous microaxial pump), surgical LV vent, atrioseptostomy, or a combination of ECMO + Impella (ECMELLA). From each of the included studies, the proportion of patients under any form of LV unloading, along with its specific modality (if mentioned), was extracted. Critical care echocardiography data included quantitative variables, time of measurement from ECMO start or attempted wean, the flow rate on ECMO at the time of the measurement, and the type of echocardiography machine used.

The type and quality of reported outcomes depended on the analysis framework of the initial research. For outcomes assessed for an association, the extracted data were odds ratio, hazard ratio, relative risk with confidence intervals, or data allowing calculation of those measures from a 2×2 table or means with standard deviation by groups. For outcomes related to diagnostic accuracy, either reported cut-off values with sensitivity and specificity and area under ROC curve, or data needed to calculate those measures were extracted.

When a study reported multiple cut-off points for a single parameter, thresholds were prioritized in the following hierarchy: (1) prespecified clinically established thresholds [e.g., LVEF 20–25%, LVOT-VTI 10 cm as per Aissaoui et al. (13)]; (2) guideline-recommended thresholds; (3) in the absence of both, the study-reported optimal or Youden-maximizing cut-off. This last group of thresholds was considered only for our initial analysis in cases where the prespecified threshold value was not available. A sensitivity analysis was conducted excluding the use of the data-driven cut-off values. The possibility of optimism associated with the use of data-driven thresholds was recognized.

Studies reporting continuous data [mean ± SD, or median/IQR convertible via the methods of Wan et al. (17) and Luo et al. (18)] contributed to the SMD meta-analysis. For studies reporting adjusted odds ratios or hazard ratios, the reported effect sizes were converted to log-transformed effect sizes (ln OR / ln HR) with standard errors derived from reported confidence intervals, then converted to SMD equivalents using the Chinn (19) conversion: SMD = ln(OR) × $\frac{\sqrt{3}}{\pi }$ . Where a single study reported both continuous and dichotomous data for the same parameter, continuous data were prioritized. No study contributed more than one estimate for a single parameter to a single pooled analysis. In cases of unclear or missing data, corresponding authors were contacted via email, with up to two follow-up attempts over a four-week period.

### 2.6 Risk of bias assessment

The hybrid nature of this review made it necessary to employ two methods for assessing risk of bias. Thus, the Newcastle-Ottawa Scale (NOS) was used to assess risk of bias for the correlation part of the meta-analysis (SMD and odds ratios from the 37 selected cohort studies). According to the NOS criteria, studies with a score of 7–9 were classified as high quality, 4–6 as moderate quality, and below 4 as low quality. Moreover, the QUADAS-2 instrument (20) was used for risk of bias assessment among the 18 cohort studies that participated in the meta-analysis of bivariate diagnostic accuracy (providing estimates of sensitivity, specificity, or ROC analysis). The QUADAS-2 evaluation was carried out for four domains: patient selection, index test, reference standard, and flow and timing. The results of QUADAS-2 are provided in Supplementary Table 2.

### 2.7 Statistical analysis

For the purposes of the statistical analyses, associations and diagnostic accuracy outcomes were analyzed separately. In case of associations, the effect measures used were the odds ratio with 95% confidence intervals, as well as the standardized mean difference (SMD) in the case of continuous data. When the studies provided median and interquartile range or range, means and standard deviations were estimated through the procedures described by Wan et al. (17) and Luo et al. (18). A random-effect meta-analysis was conducted utilizing the DerSimonian-Laird approach to estimate heterogeneity, with adjustment performed using the Hartung-Knapp method as a sensitivity analysis. Heterogeneity was assessed using Cochran’s Q-statistic and I^2^ measure.

To compute for the outcomes of diagnostic accuracy, a bivariate random-effects model was applied to estimate the pooled sensitivity and specificity, and SROC curves were generated using the method of Reitsma (16). The threshold effect was analyzed using Spearman’s correlation coefficient. The threshold value was derived from the available literature sources to provide a preliminary reference range.

The following prespecified subgroup analyses were conducted: timing of the echocardiography assessment (baseline, weaning trial, pre-decannulation); criteria for success of weaning (48 h, 24 h, hospital discharge); etiology of cardiogenic shock (acute myocardial infarction, post-cardiotomy, fulminant myocarditis, acute decompensation of heart failure, cardiomyopathy from arrhythmia); cannulation approach (peripheral versus central); LV unloading technique (IABP, Impella, surgical LV vent, no LV unloading). The following subgroup analyses were performed for all outcome variables that had ≥10 contributing studies (LVEF, LVOT-VTI, aortic valve opening, TAPSE). Although there were 12 studies to contribute to TDSa, insufficient information on subgroup data made the stratified analyses impossible; therefore, only the results of the pooled data were presented. Meta-regression was conducted using the following covariates: etiology (% post-cardiotomy, % AMI, % myocarditis), age, and publication year.

The sensitivity analyses that were performed involved the omission of single studies (leave-one-out), studies with a high risk of bias, prospective studies only, prespecified cut-off values (not driven by data), and fixed effects modeling. Funnel plot, Egger’s linear regression test, Begg’s rank correlation test, and Deeks’ funnel plot asymmetry test were used to test for the presence of publication bias. These tests were known to lack statistical power when there was considerable heterogeneity as well as when there was a low number of contributing studies. The certainty of the evidence regarding the primary outcomes (LVEF, LVOT-VTI, AVO, and TAPSE) was assessed using the GRADE approach, which evaluated study limitations, heterogeneity, indirectness, imprecision, and publication bias. All of these analyses were conducted using Review Manager 5.4, Stata 17.0, and R 4.3.0.

## 3 Results

### 3.1 Literature search and study characteristics

**Figure 1:**
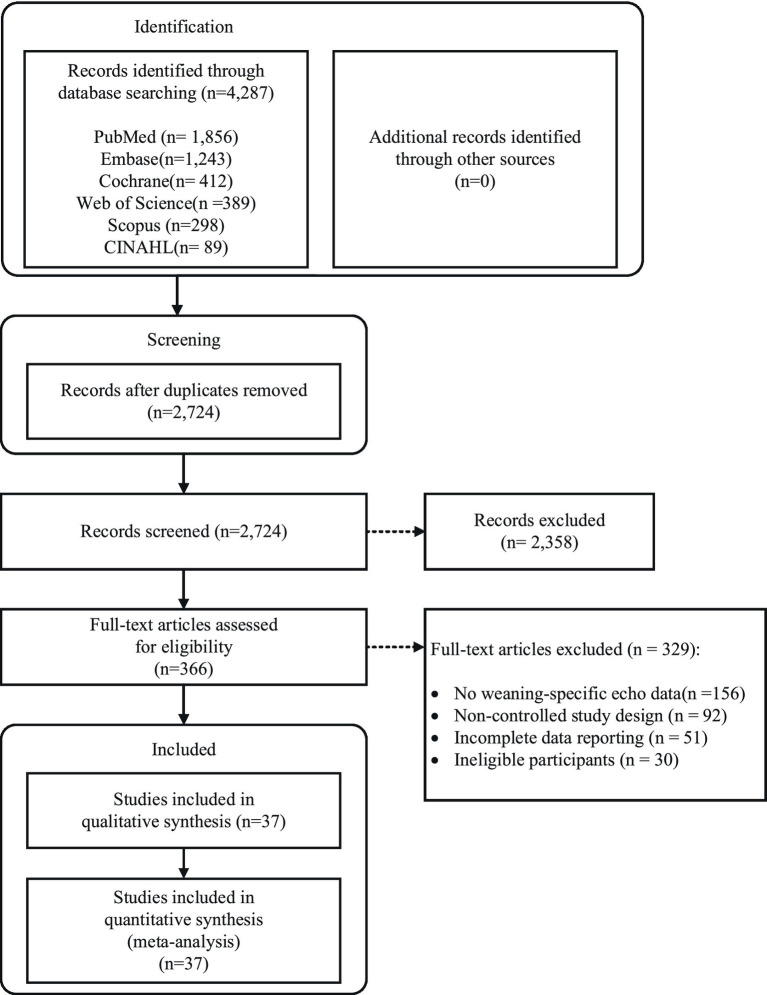
PRISMA flow diagram of study selection process.

Systematic search in all databases revealed a total of 4,287 records. After eliminating 1,563 duplicate records, 2,724 records underwent title and abstract screening with a good inter-rater reliability (Cohen’s kappa-statistic = 0.85). Of the 366 full-text articles screened, a total of 329 articles were rejected due to the lack of weaning-specific echocardiography information (*n* = 156), non-controlled study designs (*n* = 92), inadequate data presentation (*n* = 51), or non-eligible subjects (*n* = 30), leaving a total of 37 studies to be included in both qualitative synthesis and meta-analysis (Figure 1).

The trials involved a cumulative total of 3,458 patients with VA-ECMO-assisted cardiogenic shock, of which there were 1,842 successful and 1,616 unsuccessful attempts to wean the patients off VA-ECMO. The articles were published during the period from 2010 to 2025, with a majority published since 2018. In terms of geographic location, articles came from Asian centers (*n* = 17), European institutions (*n* = 13), and North American centers (*n* = 6), while there was one multi-center trial conducted internationally. Most trials had a retrospective study design (*n* = 30), followed by prospective trials (*n* = 4), and multi-center analysis of registries (*n* = 3).

Not all studies reporting a given parameter provided sufficient quantitative data for meta-analysis. For left ventricular ejection fraction, while 32 studies reported this parameter, 28 studies provided adequate data (mean ± SD or median with IQR convertible to mean ± SD) for pooled standardized mean difference calculation, and 14 studies reported diagnostic accuracy metrics (sensitivity, specificity, or ROC data) enabling inclusion in the bivariate meta-analysis. Similar attrition occurred for other parameters: LVOT-VTI was reported in 24 studies but analyzed in 22 (association) and 11 (diagnostic accuracy); aortic valve opening status was reported in 18 studies but analyzed in 16 (association) and 8 (diagnostic accuracy). This distinction between reported and analyzable studies is reflected in Tables 1, 2. Successful weaning definitions included 48-h survival (*n* = 22, 59%), 24-h survival (*n* = 6, 16%), and hospital discharge (n = 9, 24%); percentages did not sum to 100% due to rounding. When pooled estimates for LVEF were stratified by definition of successful weaning, the 48-h decannulation stratum (22 studies) yielded SMD 0.74 (95% CI 0.43–1.05, I^2^ = 76%); the 24-h stratum (6 studies) yielded SMD 0.61 (95% CI 0.28–0.94, I^2^ = 68%); and the hospital-discharge stratum (9 studies) yielded SMD 0.70 (95% CI 0.38–1.02, I^2^ = 72%). For LVEF, the respective AUCs were 0.80, 0.76, and 0.78. Although the point estimates were generally comparable, it is important to note that 24-h survival and hospital-discharge survival denote different clinical concepts—the former refers to immediate hemodynamic stability after decannulation, while the latter concerns long-term outcome. It is not advisable to consider both measures as equivalent. Estimates by strata for other variables cannot be calculated due to lack of information about outcome definitions used in individual studies.

**Table 1:** Critical care echocardiographic parameters associated with VA-ECMO weaning.

Parameter	Studies (*n*)	Patients (*n*)	SMD (95% CI)	*p* value	I^2^ (%)	Heterogeneity *P*
Left ventricular parameters						
LVEF	28	2,847	0.72 (0.44, 1.00)	<0.001	78	<0.001
LVOT-VTI	22	1,986	0.89 (0.56, 1.22)	<0.001	62	<0.001
Aortic valve opening	16	1,524	1.57 (1.01, 2.13)*	<0.001	28	0.15
TDSa (lateral s’)	12	1,156	0.52 (0.32, 0.72)	<0.001	54	0.01
MAPSE	8	687	0.54 (0.31, 0.77)	<0.001	48	0.06
E/e’ ratio	7	612	-0.56 (−0.84, −0.28)	<0.001	56	0.03
Subtotal (LV)	93	8,812	0.68 (0.52, 0.84)	<0.001	68	<0.001
Right ventricular parameters						
TAPSE	9	824	0.58 (0.32, 0.84)	<0.001	51	0.04
RVFAC	6	548	0.68 (0.34, 1.02)	<0.001	45	0.10
RV S′ (tricuspid)	5	436	0.48 (0.24, 0.72)	<0.001	38	0.17
3D RVEF	3	245	0.86 (0.43, 1.29)	<0.001	42	0.18
Subtotal (RV)	23	2,053	0.62 (0.38, 0.86)	<0.001	45	0.08
Advanced parameters						
LV GLS	4	312	0.62 (0.28, 0.96)	<0.001	52	0.10
t-IVT	3	198	-0.72 (−1.02, −0.42)	<0.001	35	0.21
Subtotal (Advanced)	7	510	0.45 (0.18, 0.72)	0.001	42	0.12

SMD, standardized mean difference; CI, confidence interval; LVEF, left ventricular ejection fraction; LVOT-VTI, left ventricular outflow tract velocity-time integral; TDSa, tissue Doppler lateral mitral annular systolic velocity; MAPSE, mitral annular plane systolic excursion; TAPSE, tricuspid annular plane systolic excursion; RVFAC, right ventricular fractional area change; RV S′, right ventricular tricuspid annular systolic velocity; 3D RVEF, three-dimensional right ventricular ejection fraction; LV GLS, left ventricular global longitudinal strain; t-IVT, total isovolumic time. *Log-transformed odds ratio presented as SMD equivalent. Subtotal counts within each category include overlapping patient contributions, as individual patients frequently contributed data to multiple parameters within the same study (total unique patients = 3,458). No cross-parameter “overall” pooled effect is reported, as such pooling would violate the assumption of independence and conflate physiologically distinct indices.

**Table 2:** Diagnostic performance and provisional cutoff values for weaning prediction (exploratory reference ranges).

Parameter	Studies (*n*)	Cutoff range	Common cutoff	Pooled sensitivity (95% CI)	Pooled specificity (95% CI)	AUC (95% CI)	LR+	LR−	DOR	Provisional threshold
LVEF (%)	14	20–30	25	0.78 (0.71–0.84)	0.68 (0.58–0.76)	0.79 (0.75–0.83)	2.44	0.32	7.56	≥20–25%
LVOT-VTI (cm)	11	10–12	10	0.82 (0.74–0.88)	0.74 (0.65–0.81)	0.85 (0.81–0.88)	3.15	0.24	12.88	≥10 cm
Aortic Valve Opening	8	Binary	Present	0.86 (0.78–0.92)	0.79 (0.68–0.87)	0.88 (0.82–0.93)	4.10	0.18	23.14	Consistently open
TDSa (cm/s)	7	6–8	6	0.76 (0.66–0.84)	0.72 (0.61–0.81)	0.81 (0.76–0.86)	2.71	0.33	8.21	≥6 cm/s
TAPSE (mm)	6	15–19	17	0.74 (0.63–0.83)	0.66 (0.54–0.76)	0.76 (0.70–0.81)	2.18	0.39	5.54	≥17 mm
RVFAC (%)	5	25–35	30	0.71 (0.59–0.81)	0.64 (0.52–0.75)	0.74 (0.67–0.80)	1.97	0.45	4.35	≥30%
E/e’ ratio	4	12–15	14	0.68 (0.55–0.79)	0.70 (0.57–0.81)	0.73 (0.66–0.79)	2.27	0.46	4.96	<14

LVEF, left ventricular ejection fraction; LVOT-VTI, left ventricular outflow tract velocity-time integral; TDSa, tissue Doppler lateral mitral annular systolic velocity; TAPSE, tricuspid annular plane systolic excursion; RVFAC, right ventricular fractional area change; LR+, positive likelihood ratio; LR-, negative likelihood ratio; DOR, diagnostic odds ratio; AUC, area under the summary receiver operating characteristic curve. Diagnostic accuracy classification: excellent (AUC ≥0.90), good (0.80–0.89), moderate (0.70–0.79), poor (<0.70). These cut-off values represent provisional, exploratory reference ranges synthesized across heterogeneous studies and should not be interpreted as firm clinical decision thresholds. They require prospective validation before being incorporated into standardized weaning protocols.

### 3.2 Quality assessment of included studies

**Figure 2:**
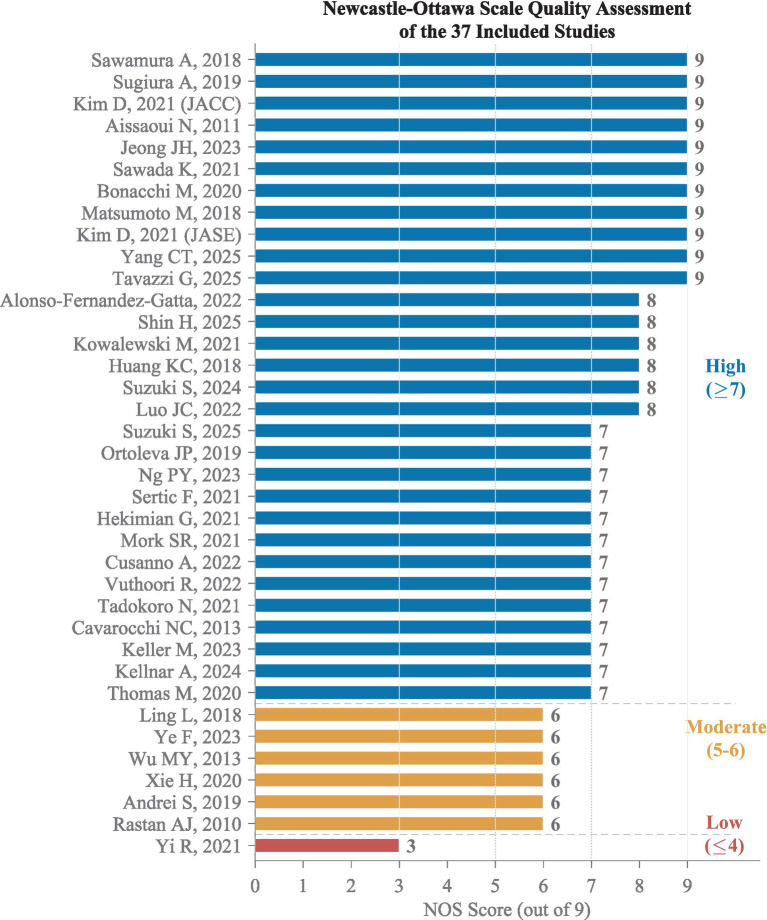
Newcastle-Ottawa scale quality assessment of the 37 included studies.

The robustness of methodology in the 37 cohort studies included was evaluated using the Newcastle-Ottawa Scale, which evaluates the methodological quality of studies in the realm of observational studies, using three criteria. The results of detailed quality assessment of each of the studies enlisted in this review are presented in Figure 2. The median NOS score was 7 stars (interquartile range 6–8) out of a maximum of 9 stars. Among the 37 studies, 30 studies (81.1%) were classified as high quality with scores of 7 or above, 6 studies (16.2%) were rated as moderate quality with scores between 5 and 6 studies (16.2%) were rated as moderate quality with a score of 6, and 1 study (2.7%) was considered low quality with a score of 3.

With regard to the selection domain, twenty-two studies (59.5%) received a full score for the VA-ECMO population’s representativeness and the selection of the comparison groups based on weaning success and the VA-ECMO population’s exposure to critical care echocardiography via standardized imaging studies carried out by experienced operators. In the domain of comparability, twenty-four studies (64.9%) showed adequate adjustment for known confounding variables: patient age, type of cardiogenic shock, and pre-procedure left ventricular function and the length of mechanical support before VA-ECMO for studies with lower scores tended to not account for the hemodynamic variables and co-interventions of intra-aortic balloon pump insertion. In the outcome domain, twelve studies (32.4%) received full scores, reflecting precise and biologically meaningful definitions of successful weaning with sufficient follow-up duration. Most of the studies (*n* = 24, 64.9%) were scored partially, mostly because of their retrospective design in terms of outcomes, not blinded to critical care echocardiographic results. The body of evidence was predominantly plagued by studies being retrospective in design (*n* = 30, 81.1%) and single-center studies (*n* = 31, 83.8%).

### 3.3 Meta-analysis of association between critical care echocardiographic parameters and weaning success

Left ventricular ejection fraction was identified as the parameter that was most commonly measured in 28 studies including 2,847 patients. The pooled standardized mean difference in LVEF between successful and unsuccessful weaning groups was 0.72 (95% CI: 0.44–1.00, *p* < 0.001), indicating that patients achieving successful weaning demonstrated significantly higher LVEF values. There was a large degree of heterogeneity between studies (I^2^ = 78%, *p* < 0.001), which could be due to variations in the time points of measurement, flow rates in ECMO during the time of assessment, and weaning criteria used in the studies included.

Left ventricular outflow tract velocity-time integral demonstrated a consistent association with weaning success across 22 studies including 1,986 patients, with a pooled SMD of 0.89 (95% CI: 0.56–1.22, *p* < 0.001) and moderate heterogeneity (I^2^ = 62%, *p* < 0.001). The results on aortic valve opening were available in 16 studies. In patients with a fixed aortic valve opening with decreased ECMO blood flow, the strongest result was observed for weaning success (SMD = 1.57, 95% CI: 1.01–2.13, *p* < 0.001), with low heterogeneity (I^2^ = 28%, *p* = 0.15). The Tissue Doppler Imaging parameter Systolic annular velocity (TDSa) was available in 12 studies with an SMD of 0.52 (95% CI: 0.32–0.72, *p* < 0.001, I^2^ = 54%). Mitral annular plane systolic excursion demonstrated similar predictive value with SMD of 0.54 (95% CI: 0.31–0.77, *p* < 0.001, I^2^ = 48%) based on 8 studies. The E/e’ ratio showed an inverse association with weaning success (SMD = -0.56, 95% CI: −0.84 to −0.28, *p* < 0.001, I^2^ = 56%), indicating that lower filling pressures were favorable for successful weaning. The pooled effect for left ventricular parameters yielded an SMD of 0.68 (95% CI: 0.52–0.84, I^2^ = 68%, *p* < 0.001).

**Figure 3:**
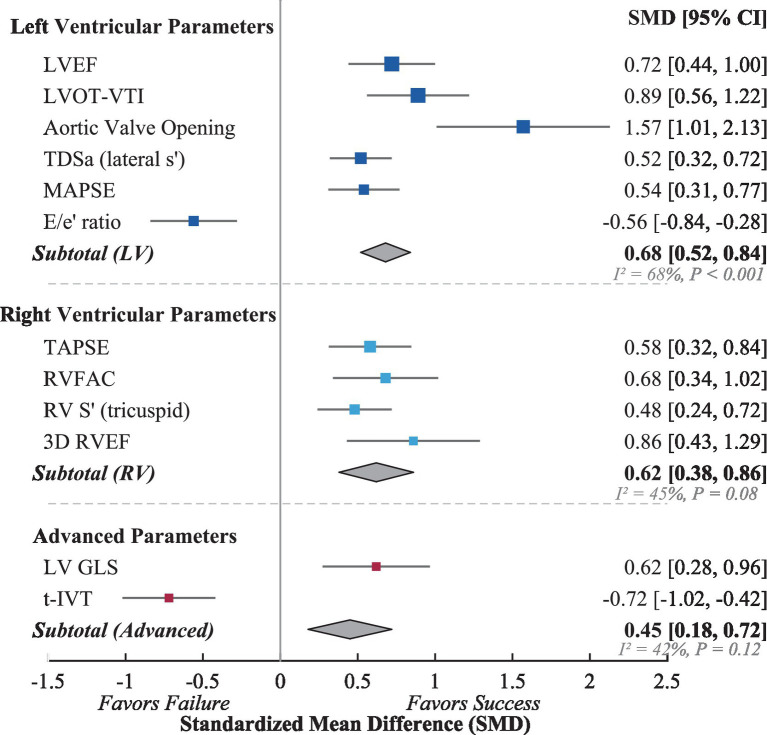
Forest plot of echocardiographic parameters for VA-ECMO weaning prediction. Note: No cross-parameter overall pooled SMD is presented. Pooling across physiologically distinct parameter categories would violate the independence assumption and double-count patients. Domain-level subtotals (LV, RV, advanced) are reported separately.

Right ventricular parameters showed moderate associations with weaning success, with a pooled RV subtotal SMD of 0.62 (95% CI: 0.38–0.86, I^2^ = 45%, *p* = 0.08). Among individual indices, three-dimensional RVEF demonstrated the largest effect size (SMD = 0.86, 95% CI: 0.43–1.29), though derived from only three studies. TAPSE (SMD = 0.58) and RVFAC (SMD = 0.68) showed comparable predictive associations; detailed estimates for all right ventricular parameters are presented in Table 1. Advanced critical care echocardiographic parameters including left ventricular global longitudinal strain (SMD = 0.62, 95% CI: 0.28–0.96, I^2^ = 52%) and total isovolumic time (SMD = −0.72, 95% CI: −1.02 to −0.42, I^2^ = 35%, indicating shorter t-IVT favored success) showed promising predictive value, with a pooled effect of 0.45 (95% CI: 0.18–0.72, I^2^ = 42%, *p* = 0.12). Instead of calculating a pooled estimate for each cross-parameter value that would improperly pool physiologically dissimilar indices and double-count patients, specific parameter estimates and domain estimates (LV, RV, advanced) are reported individually (Table 1; Figure 3).

### 3.4 Diagnostic accuracy of critical care echocardiographic parameters for predicting successful weaning

**Figure 4:**
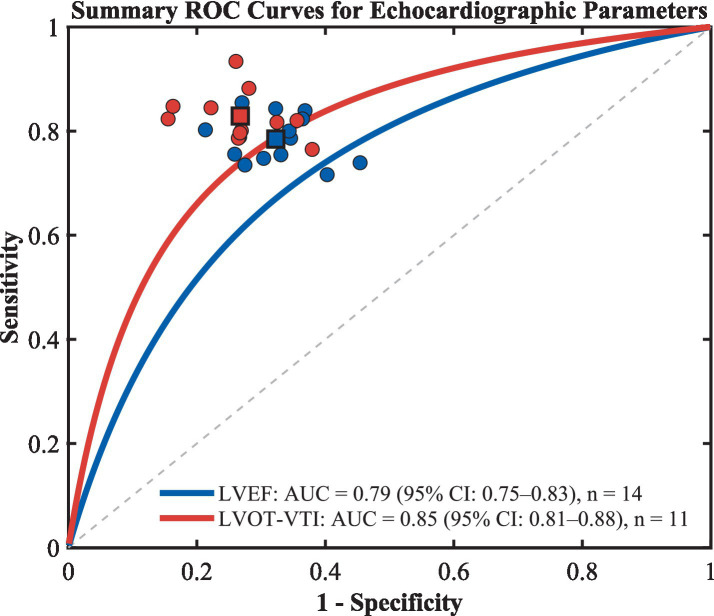
SROC curves for LVEF and LVOT-VTI in predicting VA-ECMO weaning.

Having established the association between echocardiographic parameters and weaning outcomes, the diagnostic accuracy of these parameters was further evaluated. Among the 37 studies that have been used in this systematic review, 18 have provided enough data regarding their diagnostic accuracy, such as sensitivity and specificity, to enable a random effects analysis. The remaining 19 have only given results in terms of association and not enough to construct receiver operating curves. This means that sensitivity and false positive rates are moderately correlated since their Spearman’s rho coefficient was 0.42, and *p* = 0.08. Left ventricular ejection fraction, reported in 14 studies with diagnostic accuracy data, demonstrated pooled sensitivity of 0.78 (95% CI: 0.71–0.84) and pooled specificity of 0.68 (95% CI: 0.58–0.76), yielding an area under the summary ROC curve of 0.79 (95% CI: 0.75–0.83), which corresponds to moderate diagnostic accuracy (Figure 4). The positive likelihood ratio was 2.44 (95% CI: 1.82–3.27) and negative likelihood ratio was 0.32 (95% CI: 0.24–0.44), with a diagnostic odds ratio of 7.56 (95% CI: 4.42–12.93). Left ventricular outflow tract velocity-time integral exhibited superior diagnostic performance based on 11 studies, with pooled sensitivity of 0.82 (95% CI: 0.74–0.88) and specificity of 0.74 (95% CI: 0.65–0.81), resulting in an AUC of 0.85 (95% CI: 0.81–0.88), indicative of good predictive accuracy.

**Figure 5:**
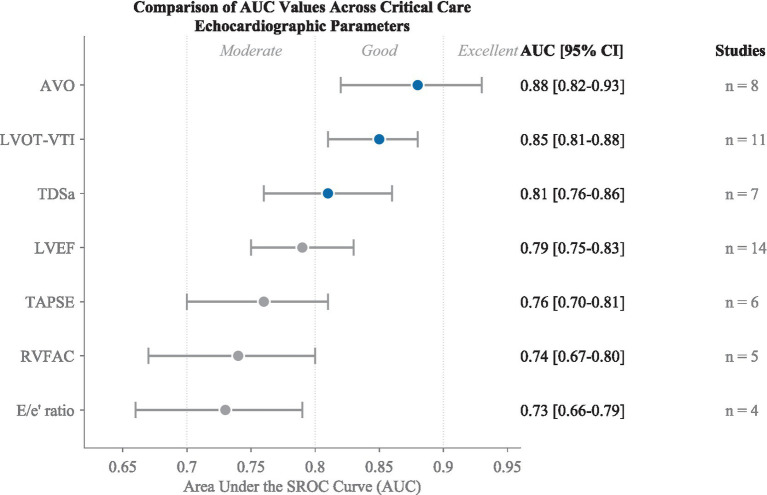
Comparison of AUC values across critical care echocardiographic parameters.

Comparison of AUC values revealed a clear hierarchy of diagnostic performance (Figure 5). Aortic valve opening status had the highest discriminative power with the largest AUC of 0.88 (95% CI 0.82–0.93), followed by LVOT-VTI with AUC of 0.85, lateral mitral annular systolic velocity by tissue Doppler echocardiography with AUC of 0.81 (95% CI 0.76–0.86), and LVEF with AUC of 0.79. Right ventricular parameters including TAPSE (AUC = 0.76, 95% CI: 0.70–0.81) and RVFAC (AUC = 0.74, 95% CI: 0.67–0.80) showed moderate diagnostic accuracy. The metrics of accuracy of the full set of diagnoses and the suggested levels of the clinical cutpoints are shown in Table 2. The systematic integration of the values mentioned in the cited studies revealed a range of 20–30% for LVEF cutpoints, wherein 25% was used most, while the cutpoints of 10–12 cm for LVOT-VTI ranged predominantly at 10 cm, which was consistent with the historic value defined by the study of Aissaoui et al. For a complete parameter-level overview integrating association and diagnostic accuracy data, readers are directed to consult Tables 1, 2 in conjunction.

### 3.5 Subgroup analysis, sensitivity analysis, and publication bias assessment

**Figure 6:**
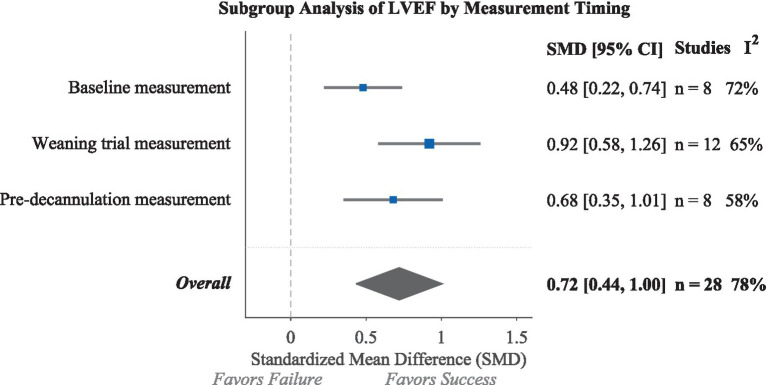
Subgroup analysis of LVEF by measurement timing (baseline, weaning trial, pre-decannulation), showing standardized mean differences with 95% confidence intervals, number of contributing studies, and heterogeneity (I^2^) for each subgroup.

Subgroup analyses were further performed for the purpose of seeking potential source of heterogeneity in left ventricular ejection fraction and successful weaning outcome. The results revealed that trials measuring LVEF during weaning trials produced a greater pooled SMD (0.92, 95% CI: 0.58–1.26, I^2^ = 65%) than those carried out at baseline (0.48, 95% CI: 0.22–0.74, I^2^ = 72%) and those done just before decannulation (0.68, 95% CI: 0.35–1.01, I^2^ = 58%), and there was a statistically significant difference between the subgroups (*p* = 0.03; Figure 6). When stratified by the definition of successful weaning, studies employing 48-h survival criteria yielded effect estimates (SMD = 0.78, 95% CI: 0.42–1.14; this estimate is derived from the full 28-study LVEF dataset in a subgroup comparison framework and therefore differs slightly from the stratum-restricted primary estimate of SMD 0.74 reported above) similar to those using hospital discharge as the endpoint (SMD = 0.70, 95% CI: 0.38–1.02), and no significant subgroup difference was detected (*p* = 0.68). Analysis according to cardiogenic shock etiology indicated that studies focusing on acute myocardial infarction demonstrated moderately higher effect sizes (SMD = 0.82, 95% CI: 0.48–1.16, I^2^ = 70%) than those including myocarditis (SMD = 0.65, 95% CI: 0.28–1.02, I^2^ = 68%) or post-cardiotomy populations (SMD = 0.58, 95% CI: 0.24–0.92, I^2^ = 54%), although the interaction test did not reach statistical significance (*p* = 0.42).

Subgroup analyses for additional primary parameters yielded concordant directions. For LVOT-VTI, pooled SMDs were: AMI 0.94 (95% CI 0.58–1.30, 9 studies), post-cardiotomy 0.71 (95% CI 0.38–1.04, 6 studies), fulminant myocarditis 1.02 (95% CI 0.58–1.46, 4 studies), and mixed/other 0.85 (95% CI 0.50–1.20, 3 studies), with no significant subgroup difference (*p* = 0.38). For aortic valve opening status, post-cardiotomy patients showed attenuated effect (SMD 1.14, 95% CI 0.48–1.80) compared with non-cardiotomy populations (SMD 1.72, 95% CI 1.12–2.32), though the interaction test was not significant (*p* = 0.19). For TAPSE, pooled SMDs were: AMI 0.64 (95% CI 0.34–0.94), post-cardiotomy 0.52 (95% CI 0.22–0.82), and myocarditis 0.71 (95% CI 0.30–1.12), without significant subgroup differences (*p* = 0.62). The prespecified subgroup analysis by cannulation approach (peripheral versus central) could not be performed. Although cannulation type was extracted from each study (Table 3), fewer than five studies per stratum provided parameter-level echocardiographic data disaggregated by cannulation type in a form amenable to pooled analysis. This analysis is therefore reported as not feasible and is listed as an unexecuted prespecified analysis in Supplementary File 1.

**Table 3:** Basic characteristics of included studies.

References	Country	Study design	Population	ECMO indication	Echo parameters studied	Quality score (NOS)
(3)	Italy	Retrospective cohort	Adult CS	CS	Longitudinal function, cardiac time intervals	9
(32)	Spain	Retrospective cohort	Adult CS	CS	LVEF, LVOT-VTI, TDI	8
(33)	Taiwan	Retrospective cohort	Adult CS	CS	LVEF changes	9
(34)	France	Retrospective cohort	Adult CS	CS	Multiple echo parameters	7
(35)	Japan	Retrospective cohort	Adult CS	CS	Cardiac function parameters	8
(36)	USA	Retrospective cohort	Adult CS	CS	Cardiac recovery parameters	7
(12)	Korea	Retrospective cohort	Adult refractory CS	CS	LVEF, LVOT-VTI, TDI (s’, e’)	9
(37)	China	Retrospective cohort	Adult CS	CS	Hemodynamic parameters	8
(38)	Germany	Retrospective cohort	Adult post-cardiotomy	PCS	Standardized echo assessment	7
(39)	Germany	Retrospective cohort	Adult CS	CS	Aortic arch blood flow	7
(40)	Japan	Retrospective cohort	Adult fulminant myocarditis	FM	LVEF, cardiac function	9
(41)	Germany	Retrospective cohort	Adult CS	CS	Patient-related factors	7
(42)	Italy	Prospective cohort	Adult post-cardiotomy	PCS	Multiple predictors	9
(43)	USA	Retrospective cohort	Adult CS	CS	TEE parameters	7
(44)	Japan	Retrospective cohort	Adult fulminant myocarditis	FM	Cardiac function	7
(45)	Japan	Retrospective cohort	Adult CS	CS	LVEF, LVOT-VTI, AV opening	9
(46)	Korea	Multicenter retrospective	Adult CS	CS	Multiple parameters	9
(47)	Japan	Retrospective cohort	Adult CS	CS	Predictive score model	7
(48)	Denmark	Retrospective cohort	Adult refractory HF	CS	Systematic weaning parameters	7
(13)	France	Retrospective cohort	Adult refractory CS	CS	LVEF ≥20–25%, AV opening, VTI ≥ 10 cm	9
(49)	Korea	Retrospective cohort	Adult CS	CS	LA strain	8
(50)	Hong Kong	Retrospective cohort	Adult CS	CS	Pump-controlled trial off	6
(51)	France	Retrospective cohort	Adult arrhythmia-induced CM	CS	Cardiac function	7
(52)	China	Retrospective cohort	Adult post-cardiotomy	PCS	Quantitative echo parameters	6
(14)	Korea	Retrospective cohort	Adult refractory CS	CS	RV-PA coupling (TAPSE/PASP)	9
(53)	Taiwan	Retrospective cohort	Adult CS	CS	3D RVEF	8
(23)	USA	Retrospective cohort	Adult CS	CS	Multiple predictors	7
(54)	Hong Kong	Prospective cohort	Adult CS	CS	LV strain during flow changes	7
(55)	Taiwan	Retrospective cohort	Adult AMI with collapse	AMI	Cardiac recovery	6
(56)	USA	Retrospective cohort	Adult CS	CS	Biventricular function	7
(57)	Japan	Multicenter retrospective	Adult AMI with CA	AMI + CA	Multiple predictors	9
(58)	International	ELSO registry analysis	Elderly (≥70y) CS	CS	Mortality predictors	8
(59)	China	Retrospective cohort	Adult post-cardiotomy	PCS	In-hospital mortality after weaning	6
(60)	France	Retrospective cohort	Adult CS	CS	Descending aortic VTI	6
(61)	Japan	Multicenter prospective (CHANGE PUMP)	Adult fulminant myocarditis	FM	Early prediction model	9
(62)	Germany	Retrospective cohort	Adult post-cardiotomy	PCS	Early and late outcomes	6
(63)	China	Retrospective cohort	Adult CS	CS	LV functional parameters	3

Univariable meta-regression of LVEF effect size against the proportion of post-cardiotomy patients yielded a non-significant negative coefficient (*β* = −0.42, *p* = 0.18), and regression against the proportion of myocarditis patients yielded a non-significant positive coefficient (*β* = +0.38, *p* = 0.27). For LVOT-VTI, no covariate reached statistical significance. Given the limited number of studies and the ecological nature of study-level covariates, these results should be regarded as hypothesis-generating.

Concomitant LV unloading was examined as a potential effect modifier. Among the 37 studies, 11 (29.7%) reported universal or near-universal (>80%) use of concomitant LV unloading, 16 (43.2%) reported mixed populations (20–80%), 6 (16.2%) reported no/minimal unloading (<20%), and 4 (10.8%) did not report unloading status. For LVEF, pooled SMD in studies with low unloading rates was 0.68 (95% CI 0.38–0.98), mixed populations 0.74 (95% CI 0.44–1.04), and high-unloading populations 0.80 (95% CI 0.42–1.18), with no significant subgroup difference (*p* = 0.85). Because LV unloading directly modifies loading conditions on echocardiographic parameters—particularly LVEF, LVOT-VTI, and aortic valve opening—these subgroup estimates should be interpreted cautiously.

Sensitivity analyses confirmed the stability of key results. The pooled effect size was unaffected when studies were removed one at a time (ranging 0.65 to 0.78), and similar results were obtained when studies with high risk of bias were removed (SMD 0.70, 95% CI 0.40–1.00). Results were similar when only prospective studies were included (SMD 0.68, 95% CI 0.32–1.04), and when fixed effect models were used (SMD 0.69, 95% CI 0.58–0.80).

**Figure 7:**
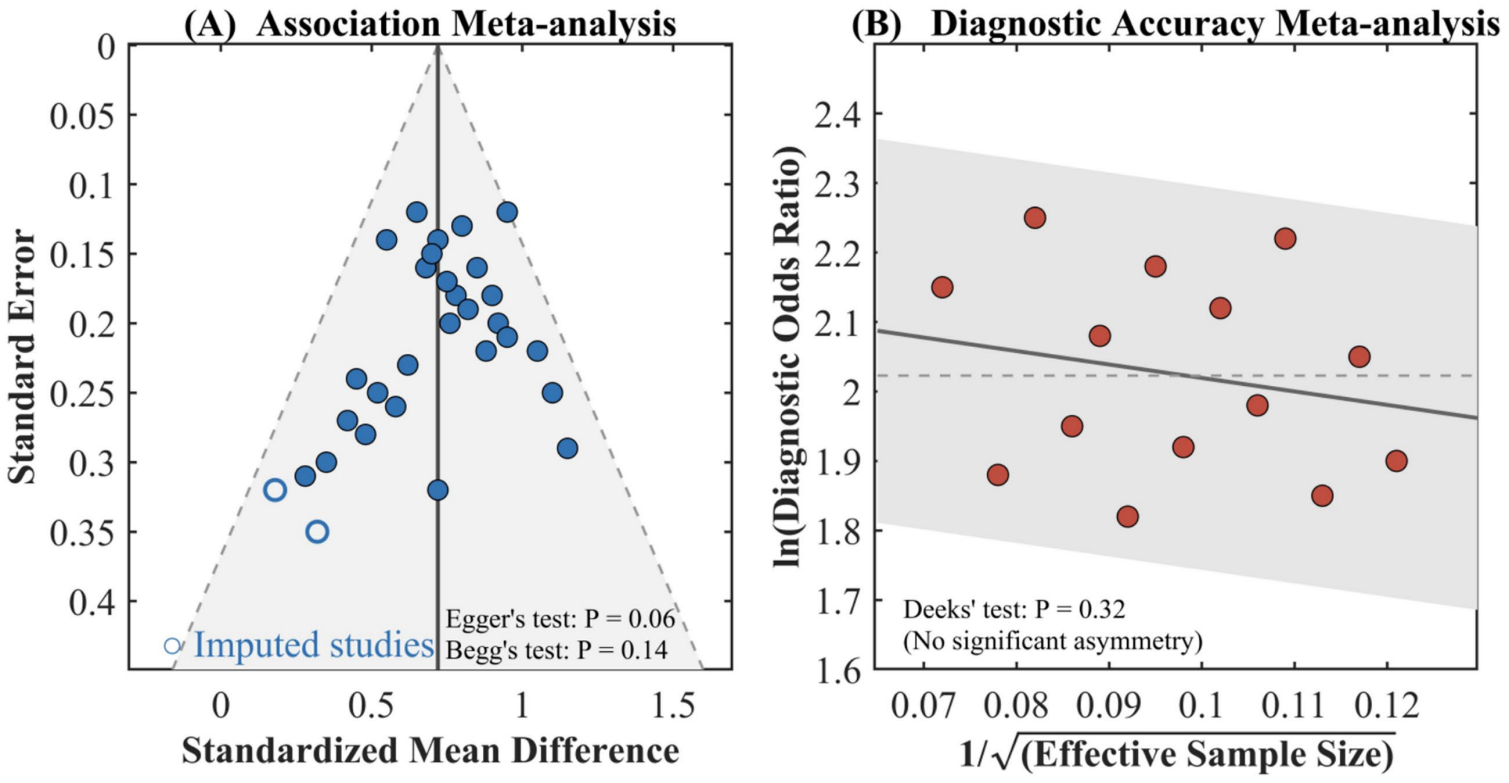
Funnel plots for publication bias assessment. **(A)** Funnel plot from the association meta-analysis. **(B)** Deeks’ funnel plot from the diagnostic accuracy meta-analysis.

Evaluations of publication bias through the use of a funnel plot revealed mild asymmetry in the association meta-analysis but relative symmetry in the diagnostic accuracy analysis, as shown in Figure 7. Egger’s regression test revealed the presence of possible small study effects in the analysis of LVEF association (intercept = 1.82, *p* = 0.06) whereas Begg’s rank correlation test was not significantly different from zero (Kendall’s *τ* = 0.18, *p* = 0.14). Deeks’ funnel plot asymmetry test revealed the absence of publication bias in the diagnostic accuracy analysis (*p* = 0.32). As a sensitivity analysis, the trim-and-fill approach suggested two missing studies and generated a new pooled effect size with SMD = 0.66 (95% CI 0.38 to 0.94). However, the trim-and-fill method is considered to be biased in the presence of high heterogeneity (I^2^ = 78% for LVEF). This is due to the assumption of the trim-and-fill approach that funnel plot asymmetry arises from publication bias rather than real difference among studies. Consequently, this finding is presented as descriptive information. Likewise, the lack of significance observed with Egger’s, Begg’s, and Deeks’ tests may not imply absence of publication bias because of low power associated with the limited number of studies.

The certainty of evidence for the four primary outcomes was assessed using the GRADE approach (21). Aortic valve opening status was rated as moderate certainty, downgraded once for risk of bias owing to the predominance of retrospective single-center designs, but not further downgraded given the low statistical heterogeneity (I^2^ = 28%). LVOT-VTI was rated as low certainty, downgraded for risk of bias and moderate inconsistency (I^2^ = 62%). LVEF was rated as low certainty, downgraded for risk of bias and serious inconsistency (I^2^ = 78%). TAPSE was rated as low certainty, downgraded for risk of bias, inconsistency (I^2^ = 51%), and imprecision reflecting the limited number of studies contributing diagnostic accuracy data (n = 6). The complete GRADE summary of findings is presented in Table 4. These ratings indicate that the pooled estimates, while directionally consistent, are subject to revision as prospective multicenter data accumulate, and reinforce the exploratory framing of the reference thresholds presented in Table 2.

**Table 4:** GRADE certainty of evidence for primary echocardiographic parameters.

Parameter	No. of studies (DTA/Assoc.)	Risk of bias	Inconsistency	Indirectness	Imprecision	Publication bias	Overall certainty
Aortic valve Opening	8/16	Serious (−1)	Not serious	Not serious	Not serious	Undetected	Moderate
LVOT-VTI	11/22	Serious (−1)	Moderate (−1)	Not serious	Not serious	Undetected	Low
LVEF	14/28	Serious (−1)	Serious (−1)	Not serious	Not serious	Possible	Low
TAPSE	6/9	Serious (−1)	Moderate (−1)	Not serious	Serious (−1)	Undetected	Low

DTA, diagnostic test accuracy meta-analysis; Assoc., association (SMD) meta-analysis. GRADE certainty levels: High/Moderate/Low/Very low.

## 4 Discussion

To the best of our knowledge, this meta-analysis is the largest conducted to date, integrating both association and accuracy measures for the use of echocardiography in predicting outcomes of VA-ECMO weaning. The main finding of this study—that aortic valve opening and LVOT-VTI are better predictors of weaning outcome than the more commonly used LVEF measurement—should be analyzed in depth.

The superior predictive value of aortic valve opening status and LVOT-VTI over LVEF has a significant pathophysiological implication for assessing myocardial recovery during VA-ECMO support. The status of aortic valve opening signifies that there has been a recovery from native left ventricular function sufficiently to counteract the elevated afterload generated by retrograde arterial ECMO support (13). This variable not only had the largest AUC value but was also most strongly correlated with the success of weaning (SMD = 1.57), with relatively low heterogeneity (I^2^ = 28%). The LVOT-VTI variable provides an ancillary quantitative estimate of the forward stroke volume, and the systematic compilation of the cutoff points verifies that the value of 10 cm remains the most empirically well-established criterion for ensuring a good cardiac output recovery (13). By contrast, for the LVEF, there was moderate diagnostic accuracy with a sensitivity of 0.78 and a specificity of 0.68, which means that a proportion, a fifth, of patients satisfying the LVEF will still suffer weaning failure. It can be seen that this measure comports with the understanding that a load-sensitive indicator such as LVEF can be misled by the conditions of VA-ECMO support (12). Contemporary understanding of cardiogenic shock pathophysiology emphasizes that successful liberation requires adequate pressure generation and flow delivery, which the superior parameters more directly reflect (4). Beyond the question of which parameter performs best, the considerable heterogeneity observed across studies warrants careful examination. Despite the moderate accuracy of LVEF compared to other parameters with superior accuracy, it is important to note that this does not preclude its use for diagnosing left ventricular diastolic dysfunction. As mentioned above, LVEF is the most common and reproducible marker and thus serves as part of multiparametric evaluation, which is supported by contemporary consensus statements (22, 23).

The considerable heterogeneity in LVEF between studies can be explained by the subgroup analysis, in which it was found that the use of echocardiography in weaning trials had substantially larger SMDs (0.92) compared to baseline (0.48) in evaluating LVEF, thus suggesting that the predictive value of these parameters is affected by the timing of the measurement. Another area of variability is represented by heterogeneity detected in definitions concerning successful weaning, yet subgroup analyses found no difference in effect size between survival criteria for survival at 48 h (SMD = 0.78) and discharge from hospital (SMD = 0.70). Efforts from the Shock Academic Research Consortium to establish consensus definitions concerning the severity level in cardiogenic shock are recent and welcome, and it would be essential to establish the same for VA-ECMO weaning (24).

The findings broadly align with recent guidance from Sinha et al. (22) on cardiogenic shock management and the Rong et al. multisociety expert consensus on perioperative echocardiographic weaning (23), the ASE Updated Recommendations for imaging of patients with temporary mechanical support (7), and the AHA Scientific Statement on escalating and de-escalating temporary mechanical circulatory support (6), all of which emphasize dynamic, multiparametric assessment during flow-reduction trials rather than reliance on any single static threshold. The pragmatic weaning framework of Randhawa et al. (8) and the foundational JACC Scientific Expert Panel guidance of Guglin et al. (5) similarly advocate for individualized, echocardiography-integrated decision-making in the context of VA-ECMO management. Consistent with Tavazzi et al. (3), longitudinal-function indices (TDSa, MAPSE, GLS) emerge as meaningful adjuncts, though the number of contributing studies remains smaller than for LVEF. Importantly, Tavazzi et al. (3) further demonstrated in multivariable analysis that LVEF was not an independent predictor of weaning success (*p* = 0.230), a finding that reinforces the provisional framing of LVEF-based thresholds adopted throughout this manuscript and supports multiparametric rather than LVEF-centric weaning assessment. Kim et al.’s demonstration that RV-PA coupling carries incremental prognostic information (14) is concordant with the moderate diagnostic accuracy observed for conventional RV indices (TAPSE, RVFAC) alone, supporting the view that coupling parameters—not captured comprehensively in the available studies—should be a priority for future work. The more cautious conclusions of Charbonneau et al. (9) regarding threshold reliability are consistent with the provisional framing adopted throughout this manuscript.

Unlike conventional ECMO for respiratory failure, VA-ECMO is not a clinical syndrome but comprises diverse groups with different pathophysiology: fulminant myocarditis in young people without heart disease and with good prospects of recovery; myocardial infarction, with recovery depending mostly on successful revascularization; post-cardiotomy shock, with the combined factors of inflammatory response to surgery, reperfusion injury, and varying baseline function; and chronic decompensated heart failure, where “weaning success” may translate into being “bridged” to LVAD/transplantation. The landmark thresholds of Aissaoui et al. (13) (LVEF 20–25%, LVOT-VTI ≥ 10 cm) are derived in a non-cardiotomy cohort and their applicability to post-cardiotomy patients has not been prospectively validated. Based on the subgroup analysis, smaller effect sizes for some parameters are found in the post-cardiotomy subgroup. It is emphasized that the derived cutoffs may not be applicable to all other etiological subgroups unless further subgroup analyses are conducted. These values must be interpreted in light of the patient’s underlying shock etiology and cannulation technique.

Simultaneous mechanical LV unloading through IABP, Impella, surgical vent, atrioseptostomy, or the more prevalent ECMELLA setup effectively changes the loading environment under which the echocardiographic metrics are obtained. ECMELLA, in particular, is known to be an active unloading device for the left ventricle, and it may result in obtaining false LVEF and LVOT-VTI readings. In contrast, VA-ECMO unilaterally raises the afterload and, as a consequence, may falsely lower LVEF in a recovering heart. Importantly, none of the analyzed studies distinguished between weaning threshold values depending on the unloading state during assessment, and this constitutes a major limitation. It is highly recommended to use the suggested syntheses with regard to ECMO flow, unloading device activity and output, and the stage of weaning trial.

An important conceptual distinction that needs to be considered is that of weaning success and post-weaning prognosis. In many of these studies, ‘successful weaning’ has been variably defined as short-term survival after decannulation (48 h in 59% and 24 h in 16% of studies), without necessarily implying long-term survival or clinical success. In addition, it would be valuable to tease apart studies that predict technical success of decannulation, or the ability to maintain hemodynamic stability shortly after decannulation, from those that predict subsequent survival or clinical outcome. The echocardiographic parameters used in these studies are mainly those of myocardial contractile function at the time of weaning, although their ability to predict long-term outcome measures such as hospital discharge or readmission rates needs further investigation. In fact, future studies would benefit from using more standardized and clinically relevant outcome measures that go beyond short-term hemodynamic stability.

Although the above discussion has been focused on LV parameters, the inclusion of right ventricular functional assessment as an important finding in this meta-analysis is worthy of consideration. It is evident from the pooled estimates of this meta-analysis that the discriminative power of tricuspid annular plane systolic excursion and right ventricular fractional area change, as determined by the area under the receiver operating characteristic curve, is only moderate. This is probably because of the limited data available and the limitations of these conventional methods of evaluating right ventricular function. It is evident that TAPSE and RVFAC can only provide a partial understanding of the complex interaction between right ventricular contractility and pulmonary vascular load, i.e., RV-pulmonary artery coupling, which is known to be a key determinant of weaning failure. These conventional methods of evaluating right ventricular function cannot possibly capture the complex, multifaceted nature of right ventricular function in the context of VA-ECMO, in which changes in pulmonary vascular resistance, left atrial pressures, and biventricular interdependence can all play a significant role. Kim and associates have shown that right ventricular pulmonary artery coupling can independently predict success of weaning and can provide incremental value over assessment of left ventricular function alone (14). Right heart failure following successful left ventricular recovery represents a significant mechanism of weaning failure that left-sided parameters alone cannot anticipate (25). This consideration takes on special meaning, especially in the context of the increased use of left ventricular unloading, since mechanical unloading devices may have differing effects on biventricular loading conditions (26). There has been a recent meta-analysis that has found differing effects between unloading modalities in regard to biventricular function, which highlights the need for a thorough echocardiographic assessment (27). Future studies should prioritise RV-PA coupling indices (e.g., TAPSE/PASP ratio) and three-dimensional RV volumetric analysis as parameters that appeared in included studies but could not be pooled due to insufficient study-level data.

The tentative ranges proposed by this meta-analysis may provide clinicians with descriptive insights regarding weaning assessment. In this respect, the meta-synthesis showed that LVEF 20–25%, LVOT-VTI ≥ 10 cm, lateral mitral annular s’ ≥ 6 cm/s, TAPSE ≥17 mm, and RVFAC ≥30% are the most frequently reported reference values. Diagnostic odds ratios also provided a clinical viewpoint in which aortic valve opening (DOR = 23.14) and LVOT-VTI (DOR = 12.88) showed higher discrimination potential than LVEF (DOR = 7.56), which supports a multimodal assessment over a unimodal one (28). The present study should be viewed as complementary to—rather than a replacement for—the recent systematic review by Hsu et al. (15). Indeed, the latter study had several strengths that could not be replicated in this review. For instance, Hsu et al.’s study involved protocol pre-registration, more stringent inclusion criteria limited only to prospective weaning protocol definitions, and a greater number of patients because of a narrower inclusion range. Of the 37 papers used in this meta-analysis, only a minority are not included in the review of Hsu et al.; these studies were published after the search period, analyzed parameters outside the interest area of Hsu et al. (such as 3D RVEF, LA strain, or total isovolumic time), or matched the wider eligibility criteria. When common parameter-specific estimates are available, pooled estimates show consistency within overlapping confidence intervals. The major contribution provided in the current meta-analysis lies in the diagnostic-accuracy bivariate analysis section, which provides sensitivity, specificity, likelihood ratio, and AUC estimates, as well as a descriptive synthesis of cut-off values used in the literature. The lack of protocol pre-registration may be considered a limitation of the review compared with that of Hsu et al.

A significant clinical limitation that needs to be highlighted is the lack of standardization in the definitions of weaning, the time of echocardiographic measurement, and ECMO flow conditions at the time of measurement. The studies used echocardiographic measurements under fundamentally different physiological states, either at baseline with full support, during flow reduction studies with varying degrees of reduced support, or just prior to decannulation. The use of measurements obtained under different states of support will inevitably lead to the conflation of different hemodynamic states. This is especially true in load-dependent parameters, as LVEF and LVOT-VTI are highly load-dependent and are significantly affected by afterload, preload, and associated LV unloading. Therefore, the strict use of LVEF cut-offs of 20–25% or LVOT-VTI cut-offs of ≥10 cm without consideration of the ECMO support (flow rate, cannulation site), the degree of LV unloading, and the time of measurement in relation to the weaning protocol may not be clinically meaningful. The thresholds derived from the studies and presented in this systematic review and meta-analysis need to be taken as guidelines and not as absolute cut-offs, and the echocardiographic parameters need to be interpreted within the individual patient’s hemodynamic environment and ECMO support.

Several limitations should be noted for this review. Firstly, 81.1% of studies included in this review were retrospective in nature, and 83.8% were performed in a single center only, thus raising concerns about selection bias, ascertainment bias, and insufficient external validity. Secondly, there were inconsistent criteria for defining “successful weaning” across studies—48-h decannulation (59%), 24-h survival (16%), and hospital discharge (24%), which represent distinct clinical outcomes, thus necessitating subgroup analyses which are presented in this review; however, these stratified analyses fail to address heterogeneity fully. Thirdly, the impact of LV unloading, known to affect load-dependent parameters, could not be fully explored due to incomplete reporting. Fourthly, cut-off values were often derived in a data-driven manner using Youden optimization in each study individually, potentially causing overoptimistic bias; sensitivity analyses restricted only to prespecified cut-offs showed moderate attenuation of diagnostic accuracy. Fifthly, VA-ECMO patients are a clinically heterogeneous group (acute myocardial infarction, post-cardiotomy, myocarditis, chronic decompensated heart failure, arrhythmia-induced cardiomyopathy), hence etiology-specific cut-offs cannot be recommended reliably based on the existing evidence. Sixthly, measurements were made under different ECMO flows (baseline full support, flow reduction, pre-decannulation), and no unified echocardiographic protocol was used. Seventhly, trim-and-fill and small-study effects tests are notoriously unreliable under high heterogeneity and moderate number of studies, and thus publication bias evaluations should be considered with caution. Eighthly, this review was not registered prospectively with PROSPERO, which represents a deviation from PRISMA 2020 best practices; however, the complete *a priori* protocol has been publicly shared in Supplementary File 1. Ninthly, individual patient data were unavailable, thus preventing further exploration of patient-level effect modifiers. Tenthly, advanced parameters (3D RVEF, GLS, LA strain, total isovolumic time, myocardial work, RV-PA coupling) are under-represented in the current literature, and their comparative value here should be viewed tentatively. Lastly, while NOS was used as the primary tool for assessing risk of bias in line with the observational cohort design of most studies included in this review, QUADAS-2 was also utilized for the subset of diagnostic accuracy studies; in relation to bivariate DTA findings, QUADAS-2 evaluations should be considered more relevant. Evidence is also still limited for advanced techniques such as speckle-tracking strain imaging (29). In addition, GRADE certainty ratings for the four primary parameters are low to moderate, driven primarily by the retrospective single-center study designs and substantial statistical heterogeneity; the exploratory reference thresholds in Table 2 should be interpreted in light of this evidence certainty.

These recommendations extend beyond the scope of the current meta-analysis data but could serve as promising hypotheses for future investigations: (1) prospective multicentric clinical trials utilizing standardized weaning protocols and definitions of outcomes; (2) examination of new parameters such as myocardial work, LA deformation, and RV–PA coupling (TAPSE/PASP ratio), which are mentioned in a small number of studies included in the meta-analysis to allow their pooled estimation; and (3) examination of AI-assisted automatic image processing, which is at an initial stage of investigation and cannot be meta-analyzed due to lack of data (30). Standardization of cardiogenic shock severity classifications would additionally support severity-stratified weaning protocols (31).

## 5 Conclusion

This meta-analysis based on 37 studies and 3,458 patients shows that critical care echocardiographic parameters have value in predicting weaning results in cardiogenic shock supported with VA-ECMO therapy. This review outlines an order of effectiveness, with aortic valve opening (AUC = 0.88) showing the highest discriminatory power, followed by LVOT-VTI (AUC = 0.85), tissue Doppler systolic velocity (AUC = 0.81), and LVEF (AUC = 0.79). The proposed reference ranges (LVEF = 20–25%, LVOT-VTI ≥ 10 cm, TAPSE ≥17 mm) in this study should be seen as speculative and are meant to guide clinical judgment but not dictate it. They are meant to be used with consideration of the patient’s underlying cause of shock, cannula placement, LV unloading state, and ECMO blood flow at the moment of assessment. An approach using multiple indices of the right and left ventricle is warranted rather than focusing on a single parameter. Multicenter prospective validation is required before these standards can be formally adopted for use as weaning criteria.

## Data Availability

The original contributions presented in the study are included in the article/Supplementary material, further inquiries can be directed to the corresponding author.
