# Supplementary material for: Meta-analysis of the predictive value of critical care echocardiography for weaning outcomes in patients with VA-ECMO-assisted cardiogenic shock
Source: Front Med (Lausanne). 2026 Jun 25;13:1835564. doi: 10.3389/fmed.2026.1835564 (PMC13346222; doi:10.3389/fmed.2026.1835564)
Supplement: Supplementary file 1 [file Table_1.DOCX]

**SUPPLEMENTARY FILE 1**

**A Priori Protocol for a Systematic Review and Meta-Analysis**

**Meta-analysis of the Predictive Value of Critical Care Echocardiography for Weaning Outcomes in Patients with VA-ECMO-Assisted Cardiogenic Shock**

**1. ADMINISTRATIVE INFORMATION**

**1.1 Title**

Meta-analysis of the Predictive Value of Critical Care Echocardiography for Weaning Outcomes in Patients with VA-ECMO-Assisted Cardiogenic Shock.

**1.2 Registration**

This systematic review was not prospectively registered in the International Prospective Register of Systematic Reviews (PROSPERO), which is acknowledged as a methodological limitation and a departure from PRISMA 2020 best practice. To mitigate the associated risk of selective reporting, this complete a priori protocol has been made publicly available as Supplementary File 1 of the published manuscript, enabling readers to compare the pre-specified methods with those reported in the final review.

**1.3 Authors**

**Miao Niu** (principal investigator): conceived the review; designed the protocol; conducted the literature search; performed statistical analyses; and drafted the manuscript.

**Yongshun Feng** (second independent reviewer): performed title and abstract screening, full-text review, data extraction, and risk-of-bias assessment; critically revised the manuscript.

The principal investigator (M.N.) takes full responsibility for the integrity of the work as a whole, from inception to published manuscript. Y.S.F. reviewed and approved the final version of the manuscript in his capacity as second independent reviewer.

**1.4 Amendments**

Any amendments to this protocol made after the commencement of data extraction, including analyses added in response to peer review, are explicitly labelled as post hoc in the published manuscript and are not considered part of the a priori plan. No such amendments will be made retrospectively to this document; the protocol represents the state of planning prior to data extraction.

**1.5 Support**

This research received no specific grant from any funding agency in the public, commercial, or not-for-profit sectors. The authors declare no conflicts of interest.

**2. INTRODUCTION**

**2.1 Rationale**

Cardiogenic shock is the most severe form of acute cardiac dysfunction, characterised by a marked reduction in cardiac output that precipitates systemic hypoperfusion and multi-organ failure. Despite advances in pharmacological and mechanical circulatory support, contemporary mortality remains between 40% and 50%. Venoarterial extracorporeal membrane oxygenation (VA-ECMO) provides temporary cardiopulmonary support for patients with refractory cardiogenic shock, and the principal therapeutic goal is successful weaning through recovery of native cardiac function.

Determining when to withdraw VA-ECMO support remains a fundamental clinical challenge. Premature decannulation risks acute haemodynamic deterioration and urgent reinitiation of extracorporeal support, whereas prolonged support increases the likelihood of bleeding, thromboembolism, infection, limb ischaemia, and haemolysis. Critical care echocardiography has emerged as the core imaging modality for assessing cardiac recovery during VA-ECMO support, with numerous parameters investigated as potential predictors of successful weaning, including left ventricular ejection fraction (LVEF), left ventricular outflow tract velocity-time integral (LVOT-VTI), aortic valve opening status, tissue Doppler–derived mitral annular systolic velocity, tricuspid annular plane systolic excursion (TAPSE), and right ventricular fractional area change (RVFAC).

The existing literature is heterogeneous with respect to the definition of successful weaning, the timing of echocardiographic measurement, and the ECMO flow conditions under which measurements are performed. Previous systematic reviews have focused predominantly on association measures, without systematically synthesising diagnostic accuracy metrics (sensitivity, specificity, and area under the summary receiver operating characteristic curve) or reported cut-off values. The present review aims to address these gaps by applying a combined analytical approach — pooling standardised mean differences alongside bivariate random-effects modelling of diagnostic accuracy — to enable parameter-level comparison and to provide a descriptive synthesis of reported cut-off values.

**2.2 Objectives**

The primary objective of this systematic review is to evaluate the predictive value of critical care echocardiographic parameters for successful weaning outcomes in adult patients receiving VA-ECMO for cardiogenic shock.

Secondary objectives are: (i) to rank echocardiographic parameters by diagnostic accuracy using summary area under the receiver operating characteristic curve (AUC); (ii) to synthesise reported cut-off values across studies to derive provisional reference ranges; (iii) to explore sources of between-study heterogeneity through pre-specified subgroup analyses and meta-regression; (iv) to examine concomitant left ventricular unloading status as a potential effect modifier; and (v) to evaluate the certainty of evidence for primary parameters using the GRADE framework.

**3. METHODS**

**3.1 Review Question**

The review question is structured using the PIRD framework (Population, Index test, Reference standard, Diagnosis of interest):

**Population:** adult patients (≥18 years) receiving VA-ECMO therapy for cardiogenic shock of any cause, including acute myocardial infarction (AMI), acute decompensated heart failure, post-cardiotomy shock, fulminant myocarditis, arrhythmia-induced cardiomyopathy, and other aetiologies.

**Index test:** critical care echocardiographic parameters measured during VA-ECMO support.

**Reference standard:** successful weaning from VA-ECMO, defined primarily as decannulation without ECMO reinsertion or initiation of durable mechanical circulatory support for ≥48 hours post-decannulation; alternative definitions (24-hour survival and survival to hospital discharge) will be analysed in pre-specified stratified analyses.

**Diagnosis of interest:** prediction of successful VA-ECMO weaning versus weaning failure.

**3.2 Eligibility Criteria**

Studies will be eligible for inclusion if they enrol adult patients (≥18 years) receiving VA-ECMO for cardiogenic shock of any aetiology and report at least one critical care echocardiographic parameter in relation to weaning outcomes. Eligible study designs comprise prospective and retrospective cohort studies, case-control studies, and diagnostic accuracy studies that report either association measures (odds ratios, hazard ratios, relative risks, or group means) or diagnostic performance at specific cut-off points. No language restrictions will be applied.

Studies will be excluded if they enrol paediatric populations (<18 years); describe venovenous ECMO for respiratory failure or extracorporeal cardiopulmonary resuscitation as the primary indication; enrol fewer than ten patients; or comprise narrative reviews, editorials, or conference abstracts without an available full text.

**3.3 Information Sources**

The literature search will be conducted across PubMed/MEDLINE, Embase, the Cochrane Central Register of Controlled Trials, Web of Science Core Collection, Scopus, and CINAHL. Clinical trial registries (ClinicalTrials.gov and the International Clinical Trials Registry Platform) will also be searched to identify completed but unpublished and ongoing research. The reference lists of included studies and of relevant systematic reviews will be manually screened to identify additional studies not retrieved by the database searches. The primary search will be conducted between 1 and 15 December 2025; the search will be updated using identical terms prior to resubmission of the manuscript.

**3.4 Search Strategy**

The search strategy will combine controlled vocabulary (MeSH and Emtree terms) with free-text terms across four conceptual domains: (i) VA-ECMO and mechanical circulatory support; (ii) cardiogenic shock and related hemodynamic compromise; (iii) weaning, liberation, and decannulation; and (iv) critical care echocardiography and related imaging techniques. Domain-specific strategies will be developed for each database. The complete, reproducible search strings for each database are presented in Supplementary Table 1.

**3.5 Study Selection**

Two reviewers (M.N. and Y.S.F.) will independently screen records in a two-step process. In the first stage, titles and abstracts will be screened against the eligibility criteria; in the second, full texts of potentially eligible studies will be retrieved and screened. Prior to formal screening, a calibration exercise will be performed on a random sample of 50 records, with a target inter-rater agreement (Cohen's κ) of at least 0.80. Discrepancies at any stage will be resolved through consensus discussion between the two reviewers. Reasons for exclusion at the full-text stage will be documented in accordance with PRISMA 2020. The study-selection process will be summarised in a PRISMA flow diagram.

**3.6 Data Extraction**

A standardised data-extraction form will be developed and piloted on a subset of included studies. Both reviewers will independently extract data, with discrepancies resolved by consensus. Corresponding authors of included studies will be contacted by electronic mail for clarification of unclear or missing data, with up to two follow-up attempts over a four-week period.

Study-level variables will include authorship, publication year, country, study design, enrolment period, and sample size. Population-level variables will include patient demographics, aetiology of cardiogenic shock, disease severity, ECMO modality (peripheral versus central cannulation), duration of ECMO support, and — with particular attention — concomitant left ventricular unloading strategies. Unloading strategies will be categorised as intra-aortic balloon pump (IABP), Impella, surgical left ventricular vent, atrioseptostomy, or ECMELLA (ECMO combined with Impella); for each study, the proportion of patients receiving any form of unloading and the specific modality used will be recorded. Echocardiographic-variable data will include the parameters quantified, the timing of measurement relative to ECMO initiation or the weaning trial, the ECMO flow rate during evaluation, and the type of echocardiographic equipment used.

For outcomes analysed as associations, extracted data will include odds ratios, hazard ratios, or relative risks with 95% confidence intervals, or sufficient raw data to reconstruct them (e.g., 2×2 tables, or group means with standard deviations). For diagnostic accuracy outcomes, extracted data will include reported cut-off points, pooled sensitivities and specificities at those cut-offs, and AUC values, or raw data enabling their calculation.

***3.6.1 Handling of Multiple Cut-off Points***

When a study reports multiple cut-off points for a single parameter, thresholds will be prioritised in the following hierarchy: (i) pre-specified clinically established thresholds (e.g., LVEF 20–25%, LVOT-VTI 10 cm per Aissaoui et al., 2011); (ii) guideline-recommended thresholds; (iii) in the absence of either of the preceding, the study-reported optimal or Youden-maximising cut-off. Category (iii) will be used in the primary analysis only when no pre-specified threshold is available. A sensitivity analysis excluding data-driven cut-offs will be performed to evaluate the potential for optimistic bias.

***3.6.2 Handling of Heterogeneous Effect Measures***

Studies reporting continuous data as mean ± standard deviation will contribute directly to the standardised mean difference (SMD) meta-analysis. Where continuous data are reported as median with interquartile range or range, means and standard deviations will be estimated using the methods of Wan et al. (2014) and Luo et al. (2018). Studies reporting adjusted odds ratios or hazard ratios will be converted to log-transformed effect sizes, with standard errors derived from reported 95% confidence intervals, and subsequently converted to SMD equivalents using the Chinn (2000) formula: SMD = ln(OR) × √3 / π. Where a single study reports both continuous and dichotomous data for the same parameter, continuous data will be prioritised. No study will contribute more than one estimate for a single parameter to a single pooled analysis.

**3.7 Risk of Bias in Individual Studies**

A two-tool approach to risk-of-bias assessment will be adopted to address the hybrid design of this review. The Newcastle-Ottawa Scale (NOS) will be applied to all included cohort studies to evaluate risk of bias for the association component of the meta-analysis; studies scoring ≥7 of 9 will be classified as high quality, 4–6 as moderate, and <4 as low. In addition, the QUADAS-2 tool will be applied to studies contributing to the bivariate diagnostic accuracy meta-analysis (i.e., those reporting sensitivity, specificity, or ROC data). QUADAS-2 will be assessed across the four domains of patient selection, index test, reference standard, and flow and timing, for both risk of bias and applicability concerns. Both tools will be applied independently by the two reviewers, with discrepancies resolved by consensus. The full QUADAS-2 assessment will be presented in Supplementary Table 2 of the manuscript.

**3.8 Data Synthesis**

Data synthesis will be conducted separately for association and diagnostic accuracy outcomes.

***3.8.1 Association Meta-Analysis***

For association outcomes, effect measures will include odds ratios with 95% confidence intervals and standardised mean differences for continuous data. Random-effects meta-analysis will be performed using the DerSimonian–Laird estimator for between-study variance, with the Hartung–Knapp adjustment applied as a sensitivity analysis given the modest number of studies per parameter. Heterogeneity will be assessed using Cochran's Q test and the I² statistic, with I² values of 25%, 50%, and 75% interpreted as low, moderate, and substantial heterogeneity respectively.

***3.8.2 Diagnostic Accuracy Meta-Analysis***

For diagnostic accuracy outcomes, bivariate random-effects models (Reitsma method) will be used to derive pooled sensitivity and specificity, with summary receiver operating characteristic (SROC) curves generated for each parameter. Threshold effect will be examined using the Spearman correlation coefficient between sensitivity and the false-positive rate. Reported threshold values will be compiled to derive provisional reference ranges, presented as exploratory rather than confirmatory estimates.

***3.8.3 Subgroup Analyses***

Pre-specified subgroup analyses will be performed for all primary parameters with at least ten contributing studies (LVEF, LVOT-VTI, aortic valve opening status, and TAPSE). Although 12 studies reported tissue Doppler mitral annular systolic velocity, insufficient reporting of subgroup-level data precluded formal stratified analysis for this parameter; results are therefore reported at the aggregate level only. The following subgroup variables are planned: timing of echocardiographic measurement (baseline, during weaning trial, pre-decannulation); definition of successful weaning (48-hour decannulation, 24-hour survival, hospital discharge); aetiology of cardiogenic shock (AMI, post-cardiotomy, fulminant myocarditis, acute decompensated heart failure, arrhythmia-induced cardiomyopathy); cannulation strategy (peripheral versus central); concomitant LV unloading status (none, IABP, Impella, surgical vent); and study risk-of-bias category.

***3.8.4 Meta-Regression***

Meta-regression will be conducted when at least ten studies are available, with the following study-level covariates: proportion of post-cardiotomy patients, proportion of AMI patients, proportion of myocarditis patients, mean patient age, and year of publication. Results will be interpreted as hypothesis-generating, given the ecological nature of study-level covariates.

***3.8.5 Sensitivity Analyses***

The following sensitivity analyses are planned: (i) leave-one-out analysis through sequential exclusion of individual studies; (ii) exclusion of studies at high risk of bias; (iii) restriction to prospective studies; (iv) restriction to pre-specified (non-data-driven) cut-off thresholds; (v) fixed-effect modelling as a comparator to random-effects results; and (vi) exclusion of studies in which means and standard deviations were estimated from medians using the Wan and Luo conversions.

**3.9 Meta-Bias Assessment**

Publication bias will be evaluated through visual inspection of funnel plots supplemented by Egger's regression test, Begg's rank correlation test, and Deeks' funnel plot asymmetry test for diagnostic accuracy outcomes. These tests are acknowledged to have reduced statistical power in the presence of substantial heterogeneity and a modest number of contributing studies; results will therefore be interpreted cautiously. The trim-and-fill method, if applied, will be reported descriptively rather than as a primary estimate, in recognition of its instability under high between-study heterogeneity.

**3.10 Confidence in Cumulative Evidence**

The certainty of evidence for each primary outcome (LVEF, LVOT-VTI, aortic valve opening status, and TAPSE) will be rated using the Grading of Recommendations Assessment, Development and Evaluation (GRADE) framework. The five GRADE domains considered will be: study limitations (risk of bias), inconsistency (heterogeneity), indirectness (population and outcome applicability), imprecision (confidence interval width and optimal information size), and publication bias. Ratings will be determined independently by the two reviewers and reconciled through consensus.

**3.11 Statistical Software**

All analyses will be performed using Review Manager version 5.4 (Cochrane Collaboration), Stata version 17.0 (StataCorp), and R version 4.3.0 (R Foundation for Statistical Computing), with the mada, metafor, and meta packages employed for the bivariate and random-effects analyses.

**4. ETHICS AND DISSEMINATION**

As this review analyses data exclusively from previously published studies without direct involvement of human participants, institutional ethics approval is not required. Findings will be disseminated through publication in a peer-reviewed journal. All pre-specified analyses will be reported regardless of statistical significance, in accordance with PRISMA 2020. Any deviations from the present protocol will be transparently reported and labelled as post hoc in the published manuscript.

**5. TIMELINE**

The planned timeline for the conduct of this systematic review is summarised below.

| Milestone | Planned date |
| --- | --- |
| Protocol finalisation | November 2025 |
| Primary literature search | 1–15 December 2025 |
| Title and abstract screening | December 2025 |
| Full-text review and eligibility assessment | January 2026 |
| Data extraction | January–February 2026 |
| Risk-of-bias assessment (NOS and QUADAS-2) | February 2026 |
| Statistical analysis and synthesis | February–March 2026 |
| Manuscript drafting | March 2026 |
| Initial submission | March 2026 |
| Updated search at resubmission | Prior to resubmission |

**6. REFERENCES**

1. Page MJ, McKenzie JE, Bossuyt PM, Boutron I, Hoffmann TC, Mulrow CD, et al. The PRISMA 2020 statement: an updated guideline for reporting systematic reviews. BMJ. 2021;372:n71.

2. McInnes MDF, Moher D, Thombs BD, McGrath TA, Bossuyt PM, PRISMA-DTA Group, et al. Preferred Reporting Items for a Systematic Review and Meta-analysis of Diagnostic Test Accuracy Studies: the PRISMA-DTA Statement. JAMA. 2018;319(4):388–396.

3. Moher D, Shamseer L, Clarke M, Ghersi D, Liberati A, Petticrew M, et al. Preferred Reporting Items for Systematic Review and Meta-Analysis Protocols (PRISMA-P) 2015 statement. Syst Rev. 2015;4(1):1.

4. Reitsma JB, Glas AS, Rutjes AW, Scholten RJ, Bossuyt PM, Zwinderman AH. Bivariate analysis of sensitivity and specificity produces informative summary measures in diagnostic reviews. J Clin Epidemiol. 2005;58(10):982–990.

5. Whiting PF, Rutjes AW, Westwood ME, Mallett S, Deeks JJ, Reitsma JB, et al. QUADAS-2: a revised tool for the quality assessment of diagnostic accuracy studies. Ann Intern Med. 2011;155(8):529–536.

6. Wells GA, Shea B, O'Connell D, Peterson J, Welch V, Losos M, Tugwell P. The Newcastle-Ottawa Scale (NOS) for assessing the quality of nonrandomised studies in meta-analyses. Ottawa Hospital Research Institute; 2013.

7. Guyatt GH, Oxman AD, Vist GE, Kunz R, Falck-Ytter Y, Alonso-Coello P, et al. GRADE: an emerging consensus on rating quality of evidence and strengths of recommendations. BMJ. 2008;336(7650):924–926.

8. Wan X, Wang W, Liu J, Tong T. Estimating the sample mean and standard deviation from the sample size, median, range and/or interquartile range. BMC Med Res Methodol. 2014;14:135.

9. Luo D, Wan X, Liu J, Tong T. Optimally estimating the sample mean from the sample size, median, mid-range, and/or mid-quartile range. Stat Methods Med Res. 2018;27(6):1785–1805.

10. Chinn S. A simple method for converting an odds ratio to effect size for use in meta-analysis. Stat Med. 2000;19(22):3127–3131.

11. Aissaoui N, Luyt CE, Leprince P, Trouillet JL, Léger P, Pavie A, et al. Predictors of successful extracorporeal membrane oxygenation (ECMO) weaning after assistance for refractory cardiogenic shock. Intensive Care Med. 2011;37(11):1738–1745.

12. DerSimonian R, Laird N. Meta-analysis in clinical trials. Control Clin Trials. 1986;7(3):177–188.

*This protocol was completed prior to the commencement of data extraction and is made publicly available as Supplementary File 1 of the published manuscript, in line with PRISMA 2020 recommendations on transparency and the mitigation of selective reporting.*
