# Supplementary material for: Meta-analysis of the predictive value of critical care echocardiography for weaning outcomes in patients with VA-ECMO-assisted cardiogenic shock
Source: Front Med (Lausanne). 2026 Jun 25;13:1835564. doi: 10.3389/fmed.2026.1835564 (PMC13346222; doi:10.3389/fmed.2026.1835564)
Supplement: Supplementary file 2 [file Table_2.DOCX]

Supplementary Material

# Supplementary Table 1. Complete Search Strategies for All Databases

| Database | Search Block | Search Terms |
| --- | --- | --- |
| PubMed/MEDLINE | #1 VA-ECMO | "Extracorporeal Membrane Oxygenation"[Mesh] OR "extracorporeal membrane oxygenation"[Title/Abstract] OR "ECMO"[Title/Abstract] OR "venoarterial ECMO"[Title/Abstract] OR "VA-ECMO"[Title/Abstract] OR "veno-arterial ECMO"[Title/Abstract] OR "extracorporeal life support"[Title/Abstract] OR "ECLS"[Title/Abstract] OR "mechanical circulatory support"[Title/Abstract] OR "cardiac ECMO"[Title/Abstract] |
|  | #2 Cardiogenic Shock | "Shock, Cardiogenic"[Mesh] OR "cardiogenic shock"[Title/Abstract] OR "cardiac shock"[Title/Abstract] OR "Heart Failure"[Mesh] OR "heart failure"[Title/Abstract] OR "acute heart failure"[Title/Abstract] OR "refractory heart failure"[Title/Abstract] OR "postcardiotomy"[Title/Abstract] OR "post-cardiotomy"[Title/Abstract] OR "Myocardial Infarction"[Mesh] OR "myocardial infarction"[Title/Abstract] OR "Myocarditis"[Mesh] OR "myocarditis"[Title/Abstract] |
|  | #3 Weaning | "Weaning"[Mesh] OR "weaning"[Title/Abstract] OR "wean"[Title/Abstract] OR "decannulation"[Title/Abstract] OR "liberation"[Title/Abstract] OR "discontinuation"[Title/Abstract] OR "withdrawal"[Title/Abstract] OR "separation"[Title/Abstract] OR "cardiac recovery"[Title/Abstract] OR "myocardial recovery"[Title/Abstract] OR "weaning outcome"[Title/Abstract] OR "weaning success"[Title/Abstract] OR "successful weaning"[Title/Abstract] OR "weaning failure"[Title/Abstract] |
|  | #4 Critical Care Echocardiography | "Echocardiography"[Mesh] OR "echocardiography"[Title/Abstract] OR "echocardiographic"[Title/Abstract] OR "Ultrasonography"[Mesh] OR "ultrasound"[Title/Abstract] OR "transthoracic"[Title/Abstract] OR "transesophageal"[Title/Abstract] OR "TTE"[Title/Abstract] OR "TEE"[Title/Abstract] OR "Stroke Volume"[Mesh] OR "ejection fraction"[Title/Abstract] OR "LVEF"[Title/Abstract] OR "left ventricular ejection fraction"[Title/Abstract] OR "velocity time integral"[Title/Abstract] OR "VTI"[Title/Abstract] OR "LVOT-VTI"[Title/Abstract] OR "aortic valve opening"[Title/Abstract] OR "aortic VTI"[Title/Abstract] OR "Echocardiography, Doppler"[Mesh] OR "tissue Doppler"[Title/Abstract] OR "TDI"[Title/Abstract] OR "mitral annular velocity"[Title/Abstract] OR "lateral s'"[Title/Abstract] OR "TAPSE"[Title/Abstract] OR "tricuspid annular plane systolic excursion"[Title/Abstract] OR "RVFAC"[Title/Abstract] OR "right ventricular fractional area change"[Title/Abstract] OR "speckle tracking"[Title/Abstract] OR "critical care echocardiography"[Title/Abstract] OR "critical care ultrasound"[Title/Abstract] OR "point-of-care ultrasound"[Title/Abstract] OR "bedside echocardiography"[Title/Abstract] OR "focused cardiac ultrasound"[Title/Abstract] OR "POCUS"[Title/Abstract] |
|  | #5 Combined | #1 AND #2 AND #3 AND #4 |
| Embase | #1 VA-ECMO | 'extracorporeal membrane oxygenation'/exp OR 'extracorporeal membrane oxygenation':ti,ab OR 'ECMO':ti,ab OR 'venoarterial ECMO':ti,ab OR 'VA-ECMO':ti,ab OR 'veno-arterial ECMO':ti,ab OR 'extracorporeal life support':ti,ab OR 'ECLS':ti,ab OR 'mechanical circulatory support'/exp OR 'mechanical circulatory support':ti,ab OR 'cardiac ECMO':ti,ab |
|  | #2 Cardiogenic Shock | 'cardiogenic shock'/exp OR 'cardiogenic shock':ti,ab OR 'cardiac shock':ti,ab OR 'heart failure'/exp OR 'heart failure':ti,ab OR 'acute heart failure':ti,ab OR 'refractory heart failure':ti,ab OR 'postcardiotomy':ti,ab OR 'post-cardiotomy':ti,ab OR 'heart infarction'/exp OR 'myocardial infarction':ti,ab OR 'myocarditis'/exp OR 'myocarditis':ti,ab |
|  | #3 Weaning | 'weaning'/exp OR 'weaning':ti,ab OR 'wean':ti,ab OR 'decannulation':ti,ab OR 'liberation':ti,ab OR 'discontinuation':ti,ab OR 'withdrawal':ti,ab OR 'separation':ti,ab OR 'cardiac recovery':ti,ab OR 'myocardial recovery':ti,ab OR 'weaning outcome':ti,ab OR 'weaning success':ti,ab OR 'successful weaning':ti,ab OR 'weaning failure':ti,ab |
|  | #4 Critical Care Echocardiography | 'echocardiography'/exp OR 'echocardiography':ti,ab OR 'echocardiographic':ti,ab OR 'ultrasound':ti,ab OR 'ultrasonography':ti,ab OR 'transthoracic':ti,ab OR 'transesophageal':ti,ab OR 'TTE':ti,ab OR 'TEE':ti,ab OR 'ejection fraction'/exp OR 'ejection fraction':ti,ab OR 'LVEF':ti,ab OR 'left ventricular ejection fraction':ti,ab OR 'velocity time integral':ti,ab OR 'VTI':ti,ab OR 'LVOT-VTI':ti,ab OR 'aortic valve opening':ti,ab OR 'aortic VTI':ti,ab OR 'Doppler echocardiography'/exp OR 'tissue Doppler':ti,ab OR 'TDI':ti,ab OR 'mitral annular velocity':ti,ab OR 'TAPSE':ti,ab OR 'tricuspid annular plane systolic excursion':ti,ab OR 'RVFAC':ti,ab OR 'right ventricular fractional area change':ti,ab OR 'speckle tracking':ti,ab OR 'critical care echocardiography':ti,ab OR 'critical care ultrasound':ti,ab OR 'point-of-care ultrasound':ti,ab OR 'bedside echocardiography':ti,ab OR 'focused cardiac ultrasound':ti,ab OR 'POCUS':ti,ab |
|  | #5 Combined | #1 AND #2 AND #3 AND #4 |
| Cochrane CENTRAL | #1 VA-ECMO | [mh "Extracorporeal Membrane Oxygenation"] OR "extracorporeal membrane oxygenation":ti,ab OR "ECMO":ti,ab OR "venoarterial ECMO":ti,ab OR "VA-ECMO":ti,ab OR "veno-arterial ECMO":ti,ab OR "extracorporeal life support":ti,ab OR "ECLS":ti,ab OR "mechanical circulatory support":ti,ab OR "cardiac ECMO":ti,ab |
|  | #2 Cardiogenic Shock | [mh "Shock, Cardiogenic"] OR "cardiogenic shock":ti,ab OR "cardiac shock":ti,ab OR [mh "Heart Failure"] OR "heart failure":ti,ab OR "acute heart failure":ti,ab OR "refractory heart failure":ti,ab OR "postcardiotomy":ti,ab OR [mh "Myocardial Infarction"] OR "myocardial infarction":ti,ab OR [mh "Myocarditis"] OR "myocarditis":ti,ab |
|  | #3 Weaning | [mh "Weaning"] OR "weaning":ti,ab OR "wean":ti,ab OR "decannulation":ti,ab OR "liberation":ti,ab OR "discontinuation":ti,ab OR "withdrawal":ti,ab OR "cardiac recovery":ti,ab OR "myocardial recovery":ti,ab OR "weaning outcome":ti,ab OR "successful weaning":ti,ab |
|  | #4 Critical Care Echocardiography | [mh "Echocardiography"] OR "echocardiography":ti,ab OR "echocardiographic":ti,ab OR "ultrasound":ti,ab OR "ejection fraction":ti,ab OR "LVEF":ti,ab OR "velocity time integral":ti,ab OR "VTI":ti,ab OR "LVOT-VTI":ti,ab OR "aortic valve opening":ti,ab OR [mh "Echocardiography, Doppler"] OR "tissue Doppler":ti,ab OR "TDI":ti,ab OR "TAPSE":ti,ab OR "RVFAC":ti,ab OR "speckle tracking":ti,ab OR "critical care echocardiography":ti,ab OR "critical care ultrasound":ti,ab OR "point-of-care ultrasound":ti,ab OR "bedside echocardiography":ti,ab OR "focused cardiac ultrasound":ti,ab OR "POCUS":ti,ab |
|  | #5 Combined | #1 AND #2 AND #3 AND #4 |
| Web of Science | #1 VA-ECMO | TS=("extracorporeal membrane oxygenation" OR "ECMO" OR "venoarterial ECMO" OR "VA-ECMO" OR "veno-arterial ECMO" OR "extracorporeal life support" OR "ECLS" OR "mechanical circulatory support" OR "cardiac ECMO") |
|  | #2 Cardiogenic Shock | TS=("cardiogenic shock" OR "cardiac shock" OR "heart failure" OR "acute heart failure" OR "refractory heart failure" OR "postcardiotomy" OR "post-cardiotomy" OR "myocardial infarction" OR "myocarditis") |
|  | #3 Weaning | TS=("weaning" OR "wean" OR "decannulation" OR "liberation" OR "discontinuation" OR "withdrawal" OR "cardiac recovery" OR "myocardial recovery" OR "weaning outcome" OR "successful weaning" OR "weaning failure") |
|  | #4 Critical Care Echocardiography | TS=("echocardiography" OR "echocardiographic" OR "ultrasound" OR "ultrasonography" OR "ejection fraction" OR "LVEF" OR "left ventricular ejection fraction" OR "velocity time integral" OR "VTI" OR "LVOT-VTI" OR "aortic valve opening" OR "aortic VTI" OR "tissue Doppler" OR "TDI" OR "mitral annular velocity" OR "TAPSE" OR "tricuspid annular plane systolic excursion" OR "RVFAC" OR "right ventricular fractional area change" OR "speckle tracking" OR "critical care echocardiography" OR "critical care ultrasound" OR "point-of-care ultrasound" OR "bedside echocardiography" OR "focused cardiac ultrasound" OR "POCUS") |
|  | #5 Combined | #1 AND #2 AND #3 AND #4 |
| Scopus | #1 VA-ECMO | TITLE-ABS-KEY("extracorporeal membrane oxygenation" OR "ECMO" OR "venoarterial ECMO" OR "VA-ECMO" OR "veno-arterial ECMO" OR "extracorporeal life support" OR "ECLS" OR "mechanical circulatory support" OR "cardiac ECMO") |
|  | #2 Cardiogenic Shock | TITLE-ABS-KEY("cardiogenic shock" OR "cardiac shock" OR "heart failure" OR "acute heart failure" OR "refractory heart failure" OR "postcardiotomy" OR "post-cardiotomy" OR "myocardial infarction" OR "myocarditis") |
|  | #3 Weaning | TITLE-ABS-KEY("weaning" OR "wean" OR "decannulation" OR "liberation" OR "discontinuation" OR "withdrawal" OR "cardiac recovery" OR "myocardial recovery" OR "weaning outcome" OR "successful weaning" OR "weaning failure") |
|  | #4 Critical Care Echocardiography | TITLE-ABS-KEY("echocardiography" OR "echocardiographic" OR "ultrasound" OR "ultrasonography" OR "ejection fraction" OR "LVEF" OR "left ventricular ejection fraction" OR "velocity time integral" OR "VTI" OR "LVOT-VTI" OR "aortic valve opening" OR "aortic VTI" OR "tissue Doppler" OR "TDI" OR "mitral annular velocity" OR "TAPSE" OR "tricuspid annular plane systolic excursion" OR "RVFAC" OR "right ventricular fractional area change" OR "speckle tracking" OR "critical care echocardiography" OR "critical care ultrasound" OR "point-of-care ultrasound" OR "bedside echocardiography" OR "focused cardiac ultrasound" OR "POCUS") |
|  | #5 Combined | #1 AND #2 AND #3 AND #4 |
| CINAHL | #1 VA-ECMO | (MH "Extracorporeal Membrane Oxygenation") OR TI "extracorporeal membrane oxygenation" OR AB "extracorporeal membrane oxygenation" OR TI "ECMO" OR AB "ECMO" OR TI "VA-ECMO" OR AB "VA-ECMO" OR TI "venoarterial ECMO" OR AB "venoarterial ECMO" OR TI "extracorporeal life support" OR AB "extracorporeal life support" OR TI "ECLS" OR AB "ECLS" OR TI "mechanical circulatory support" OR AB "mechanical circulatory support" |
|  | #2 Cardiogenic Shock | (MH "Shock, Cardiogenic") OR TI "cardiogenic shock" OR AB "cardiogenic shock" OR (MH "Heart Failure") OR TI "heart failure" OR AB "heart failure" OR TI "acute heart failure" OR AB "acute heart failure" OR TI "postcardiotomy" OR AB "postcardiotomy" OR (MH "Myocardial Infarction") OR TI "myocardial infarction" OR AB "myocardial infarction" OR TI "myocarditis" OR AB "myocarditis" |
|  | #3 Weaning | (MH "Weaning") OR TI "weaning" OR AB "weaning" OR TI "decannulation" OR AB "decannulation" OR TI "liberation" OR AB "liberation" OR TI "discontinuation" OR AB "discontinuation" OR TI "cardiac recovery" OR AB "cardiac recovery" OR TI "myocardial recovery" OR AB "myocardial recovery" OR TI "weaning outcome" OR AB "weaning outcome" OR TI "successful weaning" OR AB "successful weaning" |
|  | #4 Critical Care Echocardiography | (MH "Echocardiography") OR TI "echocardiography" OR AB "echocardiography" OR TI "echocardiographic" OR AB "echocardiographic" OR TI "ultrasound" OR AB "ultrasound" OR TI "ejection fraction" OR AB "ejection fraction" OR TI "LVEF" OR AB "LVEF" OR TI "velocity time integral" OR AB "velocity time integral" OR TI "VTI" OR AB "VTI" OR TI "LVOT-VTI" OR AB "LVOT-VTI" OR TI "aortic valve opening" OR AB "aortic valve opening" OR TI "tissue Doppler" OR AB "tissue Doppler" OR TI "TDI" OR AB "TDI" OR TI "TAPSE" OR AB "TAPSE" OR TI "RVFAC" OR AB "RVFAC" OR TI "speckle tracking" OR AB "speckle tracking" OR TI "critical care echocardiography" OR AB "critical care echocardiography" OR TI "critical care ultrasound" OR AB "critical care ultrasound" OR TI "point-of-care ultrasound" OR AB "point-of-care ultrasound" OR TI "bedside echocardiography" OR AB "bedside echocardiography" OR TI "POCUS" OR AB "POCUS" |
|  | #5 Combined | #1 AND #2 AND #3 AND #4 |
| ClinicalTrials.gov | Search Terms | Condition or disease: "cardiogenic shock" OR "heart failure" \| Intervention/treatment: "ECMO" OR "extracorporeal membrane oxygenation" OR "VA-ECMO" \| Other terms: "weaning" OR "echocardiography" OR "critical care echocardiography" OR "point-of-care ultrasound" OR "decannulation" |

Notes: Search was conducted from database inception through December 2025. No language restrictions were applied. The search strategy combined four conceptual domains: (1) VA-ECMO and mechanical circulatory support, (2) cardiogenic shock and related conditions, (3) weaning and cardiac recovery, and (4) critical care echocardiography and specific parameters.

*Abbreviations: ECMO, extracorporeal membrane oxygenation; VA-ECMO, venoarterial extracorporeal membrane oxygenation; ECLS, extracorporeal life support; LVEF, left ventricular ejection fraction; VTI, velocity-time integral; LVOT, left ventricular outflow tract; TDI, tissue Doppler imaging; TAPSE, tricuspid annular plane systolic excursion; RVFAC, right ventricular fractional area change; TTE, transthoracic echocardiography; TEE, transesophageal echocardiography.*

**Supplementary Table 2. QUADAS-2 Risk of Bias and Applicability Assessment for Studies Contributing to the Diagnostic Accuracy Meta-Analysis (n = 18)**

| No. | Study | Risk of Bias | | | | Applicability Concerns | | | Overall Risk of Bias |
| --- | --- | --- | --- | --- | --- | --- | --- | --- | --- |
|  |  | Patient Selection | Index Test | Reference Standard | Flow & Timing | Patient Selection | Index Test | Reference Standard |  |
|  |  | D1 | D2 | D3 | D4 | A1 | A2 | A3 |  |
| 1 | Aissaoui et al., 2011 (Intensive Care Med) | Low | Low | Low | Low | Low | Low | Low | Low |
| 2 | Cavarocchi et al., 2013 (J Thorac Cardiovasc Surg) | Unclear | Low | Unclear | Unclear | Low | Low | Low | Moderate |
| 3 | Matsumoto et al., 2018 (ESC Heart Fail) | Low | Unclear | Low | Unclear | Low | Low | Low | Moderate |
| 4 | Sawamura et al., 2018 (Circ J — CHANGE PUMP) | Low | Low | Low | Low | Low | Low | Low | Low |
| 5 | Huang et al., 2018 (J Am Soc Echocardiogr) | Low | Unclear | Low | Unclear | Low | Low | Low | Moderate |
| 6 | Sugiura et al., 2019 (Shock) | Low | Unclear | Low | Low | Low | Low | Low | Low |
| 7 | Ortoleva et al., 2019 (J Cardiothorac Vasc Anesth) | Low | Unclear | Unclear | Low | Low | Low | Low | Moderate |
| 8 | Kim et al., 2021 (J Am Soc Echocardiogr) | Low | Low | Low | Low | Low | Low | Low | Low |
| 9 | Kim et al., 2021 (JACC Cardiovasc Imaging) | Low | Low | Low | Low | Low | Low | Low | Low |
| 10 | Sawada et al., 2021 (ESC Heart Fail) | Low | Low | Low | Low | Low | Low | Low | Low |
| 11 | Mørk et al., 2021 (Acta Anaesthesiol Scand) | Low | Low | Low | Low | Low | Low | Low | Low |
| 12 | Alonso-Fernández et al., 2022 (Am J Crit Care) | Low | Unclear | Low | Low | Low | Low | Low | Low |
| 13 | Cusanno et al., 2022 (Sci Rep) | Low | Unclear | Low | Low | Low | Low | Low | Low |
| 14 | Ye et al., 2023 (J Cardiothorac Surg) | Low | Unclear | Unclear | Unclear | Low | Low | Low | Moderate |
| 15 | Kellnar et al., 2024 (Heliyon) | Low | Unclear | Low | Unclear | Low | Low | Low | Moderate |
| 16 | Suzuki et al., 2025 (J Card Fail) | Low | Low | Low | Unclear | Low | Low | Low | Low |
| 17 | Tavazzi et al., 2025 (Eur Heart J Cardiovasc Imaging) | Low | Low | Low | Low | Low | Low | Low | Low |
| 18 | Shin et al., 2025 (J Am Soc Echocardiogr) | Low | Low | Low | Unclear | Low | Low | Low | Low |

D1 = Patient Selection; D2 = Index Test; D3 = Reference Standard; D4 = Flow and Timing. A1–A3 = Applicability concerns for domains D1–D3 respectively. Domain D4 was assessed for risk of bias only. Overall risk of bias: Low = all four domains rated Low; Moderate = ≥1 domain rated Unclear, none rated High; High = ≥1 domain rated High.

Domain rating criteria. D1 (Patient Selection): Low = consecutive or all-eligible enrollment with explicit inclusion criteria and an appropriate patient spectrum; Unclear = enrollment method not described or retrospective without a clearly defined sampling frame; High = convenience sampling, post-hoc selection, or inappropriate exclusions. D2 (Index Test): Low = echocardiographic threshold pre-specified or derived from established reference values, and interpreting operator reported to be blinded to weaning outcome; Unclear = blinding not stated or threshold selection not described; High = threshold explicitly selected to maximise within-study discrimination after outcome ascertainment. D3 (Reference Standard): Low = successful weaning defined as decannulation without ECMO reinsertion for ≥48 hours, applied uniformly with a documented protocol; Unclear = outcome criteria inconsistently described or mixed across patients; High = reference standard unlikely to correctly classify the target condition. D4 (Flow & Timing): Low = all patients assessed at the same pre-defined phase of the weaning trial and the interval between index test and reference standard was clinically appropriate; Unclear = measurement timing varied across patients or was not consistently reported; High = substantial delay between index test and reference standard, or a substantial proportion of patients did not receive the reference standard.

The 18 studies included in this assessment were identified as those reporting sensitivity, specificity, area under the receiver operating characteristic curve, or likelihood ratios for ≥1 echocardiographic parameter. Ratings were determined independently by two reviewers (M.N. and Y.S.F.) and reconciled by consensus. Where full methodological details were not reported in the primary publication, the domain was rated Unclear rather than Low or High.
